# Noise Exposure in Palestinian Workers Without a Diagnosis of Hearing Impairment: Relations to Speech-Perception-in-Noise Difficulties, Tinnitus, and Hyperacusis

**DOI:** 10.1044/2022_JSLHR-22-00461

**Published:** 2023-02-20

**Authors:** Adnan M. Shehabi, Garreth Prendergast, Hannah Guest, Christopher J. Plack

**Affiliations:** a Manchester Centre for Audiology and Deafness, The University of Manchester, United Kingdom; b Department of Audiology and Speech Therapy, Birzeit University, Palestine; c Department of Psychology, Lancaster University, United Kingdom

## Abstract

**Purpose:**

Many workers in developing countries are exposed to unsafe occupational noise due to inadequate health and safety practices. We tested the hypotheses that occupational noise exposure and aging affect speech-perception-in-noise (SPiN) thresholds, self-reported hearing ability, tinnitus presence, and hyperacusis severity among Palestinian workers.

**Method:**

Palestinian workers (*N* = 251, aged 18–70 years) without diagnosed hearing or memory impairments completed online instruments including a noise exposure questionnaire; forward and backward digit span tests; hyperacusis questionnaire; the short-form Speech, Spatial and Qualities of Hearing Scale (SSQ12); the Tinnitus Handicap Inventory; and a digits-in-noise (DIN) test. Hypotheses were tested via multiple linear and logistic regression models, including age and occupational noise exposure as predictors, and with sex, recreational noise exposure, cognitive ability, and academic attainment as covariates. Familywise error rate was controlled across all 16 comparisons using the Bonferroni–Holm method. Exploratory analyses evaluated effects on tinnitus handicap. A comprehensive study protocol was preregistered.

**Results:**

Nonsignificant trends of poorer SPiN performance, poorer self-reported hearing ability, greater prevalence of tinnitus, greater tinnitus handicap, and greater severity of hyperacusis as a function of higher occupational noise exposure were observed. Greater hyperacusis severity was significantly predicted by higher occupational noise exposure. Aging was significantly associated with higher DIN thresholds and lower SSQ12 scores, but not with tinnitus presence, tinnitus handicap, or hyperacusis severity.

**Conclusions:**

Workers in Palestine may suffer from auditory effects of occupational noise and aging despite no formal diagnosis. These findings highlight the importance of occupational noise monitoring and hearing-related health and safety practices in developing countries.

**Supplemental Material:**

https://doi.org/10.23641/asha.22056701

Occupational noise exposure is associated with auditory and nonauditory symptoms such as noise-induced hearing loss (NIHL), temporary threshold shifts, tinnitus, hyperacusis, increased stress, cardiovascular disease, and hypertension (Basner et al., 2014; Sheppard et al., 2020). The International Standards Organization (ISO) defined maximum permissible levels of occupational noise exposure as 85–90 dB(A) L_eq_ for 8 hr per day (40 hr per week; ISO/R 1999:1971; ISO, 1971). Different developed countries have adopted different maximum permissible occupational noise exposure limits within this range (Shaikh, 1999). In developing countries, since many workers are present in the workplace for 6 days a week and 8 hr a day (i.e., 48 hr per week), a maximum permissible limit of occupational noise of 88 dB(A) L_eq_ for 8 hr per day has been proposed as a feasible and cost-effective criterion (i.e., realistic to implement) that yet meets the upper ISO limit of maximum occupational noise permissible level (Shaikh, 1999).

Permanent hearing impairment secondary to noise exposure is widely known as NIHL (D. I. Nelson et al., 2005). According to the World Health Organization (WHO), occupational noise exposure accounts for about 16% of adult disabling hearing impairment (DHI) cases worldwide, with up to 21% in some developing world subregions (Concha-Barrientos et al., 2004; D. I. Nelson et al., 2005). Similarly, a systematic review by Lie et al. (2016) estimated that about 7%–21% of hearing loss is attributable to occupational noise exposure among workers, with a significantly higher prevalence in developing than industrialized countries. This could be explained by the fact that regulations on the maximum permissible levels of occupational noise and the use of protective hearing equipment at the workplace are more strictly implemented in industrialized and developed countries (Tikka et al., 2012). The International Labor Organization investigated occupational health and safety measures in the Palestinian territories and highlighted the lack of strict implementation of occupational health and safety laws and the noncompliance with such regulations in Palestinian industries (International Labour Office, 2017, 2018). A recent study found that about 32% of industrial workers in Palestine had occupational injuries, and thus, the authors concluded that occupational health and safety measures are poorly regulated in Palestine compared to other countries (Tuhul et al., 2021).

Noise and ototoxic exposures are health and safety hazards in some workplaces (Lie et al., 2016). Alongside other etiologies such as metabolic cochlear changes; lifestyle-related factors such as smoking, alcohol intake, low socioeconomic status, dietary aspects, and general health (e.g., cardiovascular disease and diabetes); and genetic susceptibility, these factors may contribute to age-related hearing loss (ARHL; Gates & Mills, 2005; Tas, 2022; Toppila et al., 2001). At a physiologic level, both NIHL and ARHL manifest as sensorineural hearing loss due to permanent damage to the cochlear outer hair cells (OHCs) inner hair cells (IHCs), and spiral ganglion cells (Gates & Mills, 2005; Huang & Tang, 2010; E. G. Nelson & Hinojosa, 2006; Wang et al., 2002). In both NIHL and ARHL, difficulties understanding speech in noisy environments are common due to permanently elevated audiometric thresholds, worse temporal resolution, and poorer frequency selectivity (Findlay, 1976; Gates & Mills, 2005; Scheidt et al., 2010; Schorn & Zwicker, 1990).

Evidence from several animal species suggests that noise exposure and aging may damage the cochlear synapses that connect the inner hair cells with the auditory nerve well before cochlear hair cells are damaged (Kujawa & Liberman, 2009, 2015; Lin et al., 2011; Shehabi, Prendergast, & Plack, 2022; Valero et al., 2017). Low-to-medium spontaneous-rate (SR) high-threshold auditory nerve fibers (ANFs) were observed to be particularly vulnerable to this cochlear synaptopathy (CS) in guinea pigs and gerbils (Furman et al., 2013; Schmiedt et al., 1996), but not in CBA/CaJ mice, in which high-SR fibers were equally affected (Suthakar & Liberman, 2021). In older human adults, postmortem temporal bone studies presented histopathological evidence for age-related CS and ANF loss (Viana et al., 2015; Wu et al., 2019, 2021). Furthermore, middle-aged humans with a confirmed history of occupational noise exposure and with no self-reported otologic symptoms exhibited significantly fewer ANFs compared to their low-noise middle-aged counterparts (Wu et al., 2021). Low-to-medium SR ANFs may code moderate- to high-level sounds, such as speech in humans (Bharadwaj et al., 2014; Huet et al., 2016; Kujawa & Liberman, 2015). Hence, humans with CS in the absence of hair cell loss are hypothesized to exhibit SPiN difficulties without hearing threshold elevations (Plack et al., 2014).

Several behavioral lab-based studies have investigated the impact of noise exposure and aging on speech perception in noise (SPiN). Adults with NIHL and/or ARHL have been consistently observed to perform worse in SPiN tests compared to their counterparts with normal hearing (Acton, 1970; Dubno et al., 1984; Frisina & Frisina, 1997; Quist-Hanssen et al., 1978; Smoorenburg, 1992). However, no clear association has been found between lifetime noise exposure and SPiN performance in audiometrically normal young adults (for reviews, see Bramhall et al., 2019; Le Prell, 2019; and Shehabi, Prendergast, & Plack, 2022). In contrast, an age-related decline in SPiN performance among older adults with normal or near-normal audiometric profiles has been consistently documented in the literature (Babkoff & Fostick, 2017; Füllgrabe et al., 2015; S. Kim et al., 2006; Patro et al., 2021; Pichora-Fuller et al., 1995; Vermeire et al., 2016). However, this effect may not entirely be attributable to age-related CS, since other age-related factors, which were not controlled for in many of these studies, may also influence SPiN. These factors include central auditory neural degeneration (which may decrease temporal resolution; Caspary et al., 2008; Ouda et al., 2015), poorer cognitive function (Humes & Dubno, 2009; Kamerer et al., 2019), and elevated extended high-frequency (EHF) thresholds (Snell et al., 2002; Stelrnachowicz et al., 1989).

A few recent studies have examined the effects of noise exposure and aging on SPiN thresholds in audiometrically normal/near-normal adults, while controlling for potential age-related confounds, by presenting speech stimuli at low and high levels, thus independently assessing coding by high- and low-threshold ANFs, respectively (Carcagno & Plack, 2021; Johannesen et al., 2019; Prendergast et al., 2019). None of the aforementioned studies provided compelling evidence of poorer SPiN performance that could be attributed to noise-induced or age-related CS using the digits-in-noise (DIN) test, the coordinate response measure (CRM) task, or disyllabic words (presented in speech-shaped noise or the international female fluctuating masker). However, Johannesen et al. (2019) found that older adults performed significantly worse using sentences from the hearing-in-noise test, when embedded in speech-shaped noise and the international female fluctuating masker, compared to their younger counterparts. Recently, Shehabi, Prendergast, Guest, and Plack (2022) reported that a group of older adults with no diagnosis of hearing impairment and with low self-reported lifetime noise exposure (*n* = 34) exhibited worse SPiN thresholds (obtained using an online version of the DIN and CRM tests) compared to their younger counterparts (*n* = 79). The authors attempted to control for age-related cognitive decline by including a measure of cognitive function (digit span) as a covariate in their analyses.

Other pathologic symptoms such as tinnitus and hyperacusis are typically associated with unsafe noise exposure and aging (Ahmad & Seidman, 2004; H. J. Kim et al., 2015; McCormack et al., 2016; Nondahl et al., 2010; Oosterloo et al., 2021; Paulin et al., 2016; Shargorodsky et al., 2010; Tyler et al., 2014). According to Baguley et al. (2013), *tinnitus* is often defined as a phantom auditory effect that typically manifests as the perception of ringing, buzzing, or hissing sounds in the absence of external sound stimulation. *Hyperacusis* is defined as an abnormal intolerance to soft and moderate everyday environmental sounds (Baguley, 2003). Both tinnitus and hyperacusis can manifest in the absence of hearing impairment, and they tend to co-occur, with about 86% of patients with hyperacusis also reporting tinnitus (Anari et al., 1999; Baguley, 2003; Baguley et al., 2013).

Since ANFs are lost as part of CS, normal-hearing adults with CS are hypothesized to experience a higher prevalence of tinnitus and hyperacusis because of the increased central compensatory gain at the level of the brainstem (Bramhall et al., 2018; Hickox & Liberman, 2014; Schaette & McAlpine, 2011; Valderrama et al., 2018). A study found that a group of audiometrically normal adults with tinnitus exhibited significantly higher lifetime noise exposure than a strictly matched control group (Guest, Munro, et al., 2018). Moreover, musicians with normal hearing who are typically exposed to very loud music were found to have worse hyperacusis and greater tinnitus handicap compared to nonmusicians (Couth et al., 2020). Recently, Shehabi, Prendergast, Guest, and Plack (2022), who employed a similar online research protocol, reported that young adults with high lifetime noise exposure, but without a past diagnosis of hearing impairment, exhibited a higher prevalence of tinnitus and a higher risk of hyperacusis compared to their low-noise counterparts. Similarly, older adults with low lifetime noise exposure exhibited a higher prevalence of tinnitus, but not worse severity of hyperacusis, compared to their younger adult counterparts.

The current study was based on a novel approach of collecting SPiN thresholds and self-reported hearing data online from an underresearched population that is thought to be regularly exposed to unsafe levels of occupational noise. The aim was to quantify the effects of occupational noise exposure and aging on the hearing function of adults without a formal diagnosis of hearing loss in Palestine. The primary aims of the current study were to compare the effects of occupational noise exposure and aging on (a) SPiN thresholds using an online Arabic version of the DIN test, (b) self-reported hearing ability, (c) presence of tinnitus, and (d) severity of hyperacusis. The secondary aim of this study was to determine the effects of both occupational noise exposure and aging on the severity of tinnitus handicap. We hypothesized that higher occupational noise exposure and older age would be associated with (a) higher SPiN thresholds, (b) poorer self-reported hearing ability, (c) a higher proportion of participants with tinnitus, (d) greater tinnitus handicap, and (e) greater severity of hyperacusis.

## Method

This study was preregistered on the Open Science Framework before the beginning of data collection. All the hypotheses and data collection procedures are in line with the preregistered protocol (http://osf.io/xtb6e). For the statistical analyses, some aspects of the observed dataset required us to deviate from the analysis plan laid out in the preregistered protocol; all such deviations and the reasons for them are outlined below. For the sake of transparency and completeness, Supplemental Material S1 includes the statistical analyses performed strictly according to the preregistered study protocol.

### Participants

The study sample comprised 251 Palestinian adult participants (152 women) aged 18–70 years (*M*_age_ = 35.1 years, *SD* = 13.6), most of whom worked in noisy industries. Participants were recruited through online advertising and by contacting several noisy industrial employers in the West Bank of Palestine. The noisy industries from which participants were recruited included construction sites, factories, carpentries, blacksmiths, agriculture, roadworks, bakeries, nurseries, schools, and car garages. Participants had a variety of educational backgrounds. Table 1 shows the highest formal academic qualifications reported by participants. None of the participants reported past intake of ototoxic medications or recent diagnosis of ear or hearing disorders, or pathologies such as balance problems or head/ear traumas. Moreover, no currently or recently diagnosed neurological, mental health, or memory disorders were reported by any of the participants.

**Table 1.** The distribution of the highest formal academic qualifications reported by male and female participants.

Qualification	Primary school	Middle school	High school	Diploma/vocational training	Undergraduate university degree	Postgraduate university degree
Sex						
Male	2	12	9	14	38	24
Female	2	4	13	15	89	29
Total	4	16	22	29	127	53

Thirty-six participants were excluded: 21 participants had a diagnosis of hearing loss, eight had a diagnosis of neurological/memory disorders, and seven did not meet the age criteria of the study. Participants had the opportunity to read a detailed study information sheet before taking part and to ask questions by e-mail if they needed further information or clarifications. Informed consent was provided online upon participation. To thank our participants for their time and engagement in our study, a prize draw was performed at the end of the study, and four participants won online shopping vouchers. The study was approved by the University of Manchester Research Ethics Committee (ethics application reference: 2020-8884-13533).

### Online Instruments

All study instruments (see below) were incorporated into the Research Electronic Data Capture (REDCap) platform, which is a participant-friendly online research tool (Harris et al., 2009, 2019). For the current study, the platform was hosted at the University of Manchester. All study participants (*N* = 251) performed all the online instruments except for the Tinnitus Handicap Inventory (THI) and the DIN test, which were performed by a subset of 59 and 152 participants, respectively. Only participants who reported tinnitus were invited to complete the THI, and although all study participants were invited to perform the DIN task, not all completed it.

#### Otologic Health and Demographic Information

Relevant demographic and general health information was collected using a clinical and demographic online questionnaire developed by the researchers in Modern Standard Arabic (MSA; see Supplemental Material S2). Participants were asked to state/identify demographic information related to their age, sex, educational attainment, past and current employment, and contact details. Questions on otologic health covered past ear and hearing disorders including traumas to the ears, head, and neck; tinnitus; hyperacusis; balance problems; intake of ototoxic medications; and family history of hearing impairment. General health questions included past and current chronic health conditions and/or disabilities and the intake of medications.

#### Noise Exposure

An online noise exposure questionnaire written in MSA based on the Noise Exposure Structured Interview (NESI; Guest, Dewey, et al., 2018) was used to quantify both occupational and recreational noise exposure (see Supplemental Material S3). The NESI noise exposure estimation approach follows the work of Lutman et al. (2008). Given its advantages over other self-reported noise-exposure estimation instruments (Guest, Dewey, et al., 2018), the NESI (or its precursor) has been used by several auditory research studies over the past few years (Causon et al., 2020; Couth et al., 2020; Guest, Munro, Prendergast, et al., 2017; Guest, Munro, et al., 2018; Prendergast et al., 2018, 2019; Prendergast, Guest, et al., 2017; Prendergast, Millman, et al., 2017; Shehabi, Prendergast, Guest, & Plack, 2022; Shehorn et al., 2020).

The noise exposure questionnaire constitutes four sections: occupational noise, recreational noise, earphone/headphone noise, and firearm noise exposure. In each section, participants reported all noisy activities where noise levels were deemed unsafe as defined by noise levels > 80 dBA. The sound level in dBA in each noise exposure activity was estimated by asking participants to identify the vocal effort needed to maintain a conversation in that situation, or for personal listening devices, to identify their typical volume control setting (Guest, Dewey, et al., 2018). For each noisy situation, participants stated the number of years, weeks per year, days per week, and hours per day that they were exposed to the noise. Then, participants were asked to report any use of hearing protection and (if used) state their type(s) to allow for the estimation of protector attenuation. The magnitude of noise exposure in each noisy situation was determined using the following formula:

$$U=10^{\left(L-A-90\right)/10}\times \frac{T}{2080}\text{,}$$ (1)

where *U* = units of noise exposure (energy), *L* = level (dBA), A = attenuation of ear protection, and *T* = total exposure time. For each section (i.e., recreational, occupational, and firearm), the units of noise exposure were added to produce a raw noise exposure score. Participants who did not report any noisy activities in either the recreational or occupational sections were assigned a raw noise score of 0.0001 per section. This value was selected to be less than the lowest calculated raw value of noise exposure. Since the raw noise exposure scores per section were not normally distributed, the raw scores were log-transformed (log_10_[U]) to produce normally distributed noise exposure datasets. One logarithmic unit of exposure energy is equivalent to a factor of 10 of raw noise exposure. One raw noise exposure unit (*U*) equates to an exposure of 90 dB(A) of occupational noise for an entire working year of 2080 hr.

#### Cognitive Ability

To assess attention and short-term memory span, the forward and backward versions of the digit span test (Wechsler, 1997) were used. The REDCap platform was used to deliver the digit sequences visually. For each version of the test, a trial block of two digits only was presented to participants. In this trial block, each digit was presented for 1 s. The two digits were separated by a 1-s time delay. Participants were asked to remember the sequence of digits they saw on the screen and to enter the digits either forward or backward in sequence into the answer box once the digit presentation was completed.

The actual testing block used the same digit presentation duration and between-digits delay time (i.e., 1 s). Once a correct answer was entered in each trial of the testing block, the presentation of a new number sequence was automatically prompted. This number sequence included an additional digit. The highest possible number of digits that could be reached was nine. If an incorrect answer was entered, a new number sequence with the same number of digits was presented. The entry of two consecutive incorrect answers at the same number of digits led to the end of the testing block. The forward and backward tasks were performed separately such that the forward digit span version of the test was performed first. The participants' digit span scores were determined as the highest number of correctly identified digits in both versions of the test.

#### SPiN

An online Internet-based Arabic version of the DIN test was presented via a web browser. The online DIN task is composed of a carrier phrase and three digits ranging from 1 to 9 (“The digits {digit 1} {digit 2} {digit 3}”), embedded in speech-shaped background noise (Smits et al., 2004, 2006). This test is thought to reflect the health of the peripheral auditory system as it is minimally impacted by linguistic and central cognitive factors that could impact SPiN performance (Heinrich et al., 2015; Smits et al., 2013, 2004).

The target phrases (i.e., the carrier phrase and the digits) of the online Arabic DIN were articulated by a female talker in MSA, whereas the background noise (i.e., speech-spectrum shaped Gaussian noise) had the same long-term average speech spectrum as the set of Arabic digits. Participants were asked to complete the online Arabic DIN task using their personal computers (with mouse/trackball or trackpad) and their headphones or earphones, in a quiet room that had as few distractions as possible. During the test, participants were presented with an on-screen number pad that they were instructed to use for digit entry. To maximize participants' attention and engagement, animated visual feedback was presented on the screen following the entry of participants' responses showing whether the answer was correct or incorrect and their progress throughout the test.

In an attempt to reduce performance variability due to differences in the high-frequency bandwidth of participants' headphones/earphones, the digits and background noise were low-pass filtered at a knee point of 8 kHz. To ensure that participants' performance was not affected by the audibility of the target phrases or by the stimuli being uncomfortably loud, participants performed a subjective calibration block to ensure that presentation levels were both comfortable and audible. This calibration block comprised two sentences articulated in MSA presented at two levels that differed by 25 dB. Participants adjusted the volume control on their devices such that the low-level sentence was clearly audible, and the high-level sentence was comfortably loud.

The root-mean-square (RMS) level of the stimuli for the first trial in the test was set to be 20 dB above the level subjectively set by the participant for the low-level calibration sentence and 5 dB below the level of the high-level calibration sentence. Therefore, the test was designed to ensure that, even for trials with very low signal-to-noise ratios (SNRs), the digits did not become inaudible.

The test involved two phases: a 4-min practice phase and a 5-min testing phase. In both phases, a correct response was defined as 2/3 or 3/3 correctly identified digits. In the initial trial of both phases, the digits and the background noise were presented at an SNR of 0 dB. A two-down and one-up adaptive rule varied the SNR of the stimuli with four initial turnpoints (6-dB step size) and six threshold turnpoints (2-dB step size). The DIN threshold, defined as the SNR speech recognition threshold, was calculated as the mean of the threshold turnpoints.

#### Self-Reported Hearing Ability

The short form of the Speech, Spatial, and Qualities of Hearing Scale (SSQ12) was employed to assess participants' subjective hearing ability (Noble et al., 2013). The SSQ12 was forward and backward translated from English into MSA, and the translations were verified by a Palestinian registered English/Arabic translator (see Supplemental Material S4). The SSQ12 was employed in this study rather than the full version of the SSQ because it takes a shorter time to complete and was deemed to exhibit adequate validity, reliability, and sensitivity (Noble et al., 2013; Ou & Kim, 2017). The SSQ12 is composed of 12 statements, with five statements reflecting performance in the speech domain, three statements in the spatial domain, and four statements in the qualities of the hearing domain (Noble et al., 2013)

Participants were instructed to select a score from 0 to 10 for each statement using a drop-down menu. A greater score corresponded to better performance. Given that some statements may not be applicable, participants could highlight the inapplicable statements by selecting the “not applicable” option from the drop-down menu. The SSQ12 score was calculated per participant by determining the mean score of all the applicable statements that were rated.

#### Tinnitus

Participants were asked whether they had tinnitus by the definition set out by the British Tinnitus Association (Mancktelow, 2022). The tinnitus definition was as follows: “The perception of sound in the absence of any corresponding external sound. This noise may be heard in one ear, in both ears, in the middle of the head, or it may be difficult to pinpoint its exact location. The noise may be low, medium, or high-pitched. There may be a single noise or two or more components. The noise may be continuous, or it may come and go.”

An Arabic version of the THI was used to assess tinnitus severity among participants who reported tinnitus. The THI considers the impact of tinnitus physically, psychologically, socially, emotionally, and occupationally given different life situations (Barake et al., 2016; Newman et al., 1996). The THI (see Supplemental Material S5) involves 25 questions with three possible answer choices for each question: “always,” “sometimes,” or “never.” Four points, two points, and zero points were allocated to questions answered with “always,” “sometimes,” and “never,” respectively. The overall THI score (out of 100) was calculated as the sum of the individual scores of all possible 25 questions.

#### Hyperacusis

An Arabic version of the Khalfa hyperacusis questionnaire (see Supplemental Material S6), which contains 14 questions covering social, emotional, and attentional aspects, was used to determine sensitivity and intolerance to sounds (Khalfa et al., 2002; Shabana et al., 2011). Each question had three possible answer choices: “yes, quite a lot”; “yes, a little”; and “no.” Three points, two points, and zero points were allocated to each choice, respectively (per question). The final score was the mean across all 14 questions.

### Statistical Analyses

SPSS Version 26 was used to analyze the data. In the main analyses, we determined the effects of occupational noise exposure and age (as predictor variables) on (1) SPiN performance as shown by the DIN thresholds, (2) self-reported hearing ability as reflected by the SSQ12 scores, (3) presence of tinnitus, and (4) severity of hyperacusis. We used multiple linear regression models (for Aims 1, 2, and 4) and a logistic regression model (for Aim 3). In the secondary analyses, the effects of occupational noise exposure and age (as predictor variables) on the severity of tinnitus handicap (as shown by the THI scores) were determined using a linear regression model. In all primary and secondary regression models, both occupational noise exposure/occupational noise group and age were entered as predictor variables. The covariates of sex, academic attainment (as reflected by the highest qualification of formal academic training), recreational noise exposure, and cognitive function (as shown by the forward and backward digit span test scores) were accounted for in all the statistical models.

In order to determine whether collinearity between the predictor variables of occupational noise exposure and age that had undue influence on the findings of the different regression models, the Pearson correlation coefficient across both predictor variables and the variance inflation factor (VIF) for each predictor variable in each model were computed. The Pearson correlation coefficient between occupational noise exposure scores and age was .57, which suggests a moderate correlation rather than collinearity. The VIFs for both predictor variables in all models were < 10, which suggests that multicollinearity did not greatly influence the different models (Marquardt, 1970).

Occupational noise exposure scores were not normally distributed, in that 46% of participants had no exposure to occupational noise (see Supplemental Material S7). Hence, following advice from a Manchester Biomedical Research Centre biostatistician, the authors deviated from the preregistered analysis protocol, which would have treated the ages and occupational noise scores of all participants as continuous predictor variables. The inclusion of occupational noise scores as a continuous predictor variable in the regression models was deemed inappropriate given the large subset of participants with zero exposure. Instead, for each primary and secondary aim, we tested two models: one that treated the presence/absence of occupational noise exposure as a categorical variable and one that treated it as a continuous variable but excluded participants without occupational noise exposure. For both regression models, age was entered as a continuous predictor variable. The primary and secondary outcome variables and covariates remained the same as outlined in the preregistered protocol. This decision was made following a similar issue in lifetime noise data distribution (i.e., not normally distributed) reported by Shehabi, Prendergast, Guest, and Plack (2022) who performed additional analyses similar to those we describe below. For the sake of transparency and completeness, the data of the current study were also analyzed according to the statistical analysis plan outlined in the preregistered study protocol (see Supplemental Material S7).

In the first form of the regression model, participants were divided into two occupational noise groups: the not-exposed group (i.e., participants who reported no exposure to occupational noise) and the exposed group (i.e., participants who reported at least some occupational noise exposure). Multiple regression models were performed to answer the various primary and secondary research questions by comparing the different effects across these two groups, with age entered as an additional continuous predictor variable in the same model. The second form of regression model excluded the participants of the not-exposed group and established the effects of occupational noise exposure and age (as two continuous predictor variables) on the various primary and secondary outcome measures in the exposed group. The covariates of sex, academic attainment (as reflected by the highest qualification of formal academic training), recreational noise exposure, and cognitive function (as shown by the forward and backward digit span test scores) were accounted for in both forms of the alternative statistical model. Alpha level was adjusted for 16 multiple comparisons using the Bonferroni–Holm method, with a familywise error rate of < .05. Table 2 shows a summary of both regression models, including the participants, outcome and predictor variables, and covariates of each model.

**Table 2.** Summary of the main and exploratory regression models, including the participants, the outcome and predictor variables, and the covariates of each model.

Statistical model	Participants	Outcome variables	Predictor variables	Covariates
First regression model	All study participants	- DIN thresholds - SSQ12 scores - Tinnitus presence - THI scores - Hyperacusis scores	- Occupational noise exposure group - Age	- Sex - Forward digit span score - Backward digit span score - Recreational noise exposure score - Highest academic qualification
Second regression model	Participants of the exposed group only	- DIN thresholds - SSQ12 scores - Tinnitus presence - THI scores - Hyperacusis scores	- Occupational noise exposure score - Age	- Sex - Forward digit span score - Backward digit span score - Recreational noise exposure score - Highest academic qualification
Exploratory regression model	All study participants	- DIN thresholds - SSQ12 scores - Tinnitus presence - THI scores - Hyperacusis scores	- Occupational noise exposure group - Age - Occupational Noise Exposure Group × Age	- Sex - Forward digit span score - Backward digit span score - Recreational noise exposure score - Highest academic qualification

*Note.* DIN = digits-in-noise; SSQ12 = Speech, Spatial and Qualities of Hearing Scale; THI = Tinnitus Handicap Inventory.

Further exploratory multiple regression models were performed to assess the interaction between occupational noise exposure and age on (a) SPiN performance as shown by the DIN thresholds, (b) self-reported hearing ability as reflected by the SSQ12 scores, (c) the presence of tinnitus, (d) the severity of hyperacusis, and (e) the severity of tinnitus handicap (as shown by the THI scores). Occupational noise exposure group, age, and an interaction term (Occupational Noise Exposure Group × Age) were the predictor variables, whereas recreational noise exposure, sex, the highest academic qualification of participants, and their cognitive function (as reflected by the forward and backward digit span scores) were considered covariates. The contents of this exploratory model are summarized in Table 2.

## Results

In the following subsections, the outcomes of the first and second regression models given all primary and secondary outcome measures are presented. The first regression model considered all study participants by dividing them into two occupational noise exposure groups: the exposed and not-exposed groups. In this model, occupational noise exposure group and age were both predictor variables. The second regression model included participants of the exposed group only, and both occupational noise exposure and age were continuous predictor variables.

### Occupational Noise Exposure

**Figure 1.**
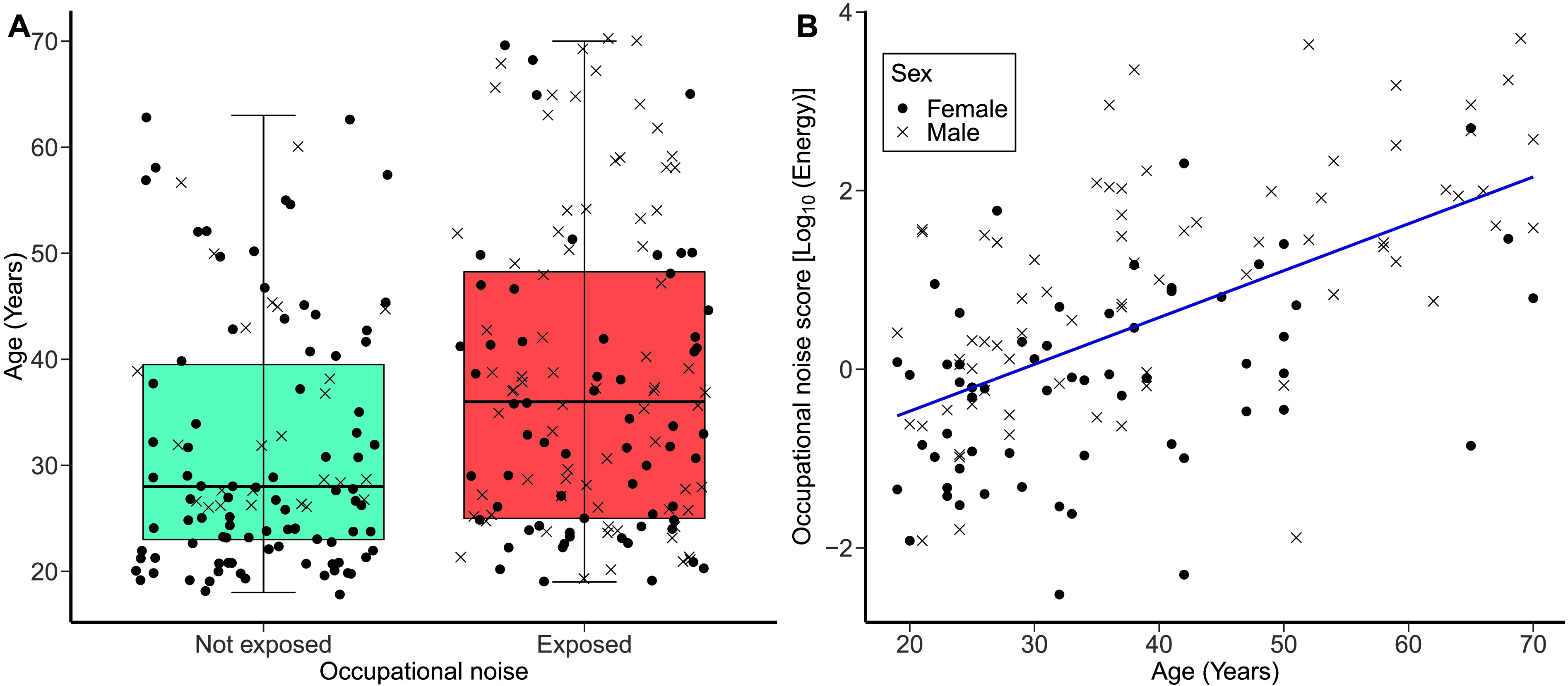
Occupational noise scores. (A) The distribution of participant age as a function of the occupational noise exposure group. The left-hand boxplot corresponds to the not-exposed group (*n* = 115), whereas the right-hand boxplot corresponds to the exposed group (*n* = 136). The upper and lower hinges represent the first and third quartiles; the thick line, the median; the upper whiskers, the highest value within 1.5 × IQR (interquartile range) of the upper hinge; and the lower whiskers, the lowest value within 1.5 × IQR of the lower hinge. (B) Occupational noise scores as a function of age for the exposed group. A best-fit regression line is drawn through the data points. For both panels, black dots and crosses correspond to individual female and male participants, respectively.

Figure 1A shows the distribution of the age of participants as a function of the occupational exposure group. The not-exposed group (*n* = 115) comprised participants with no past self-reported occupational noise exposure and who were therefore allocated an occupational noise exposure score of −4 logarithmic units (which corresponds to 0.0001 raw units of occupational noise exposure; see the Noise Exposure section). Participants who reported at least some occupational noise exposures were included in the exposed group (*n* = 136) and presented with occupational noise exposure scores ranging from −2.52 to 3.70 logarithmic units (depending on their raw scores of occupational noise as described in the Noise Exposure section).

Since age did not follow a normal distribution across both noise groups (*p* < .05 for the Kolmogorov–Smirnov test), a Wilcoxon–Mann–Whitney nonparametric test was used to compare the mean ages of participants in the groups. The participants of the exposed group were significantly older (*M*_age_ = 38.0 years, *SD* = 14.5, 95% CI [35.6, 40.5]) than those of the not-exposed group (*M*_age_ = 31.7 years, *SD* = 11.7, 95% CI [29.5, 33.9]; *U* = 9924.5, *p* < .0001).

Figure 1B illustrates the occupational noise scores (expressed in logarithmic units) of the exposed group as a function of the age of the participants. A linear regression model with age as the predictor variable and occupational noise score (expressed in logarithmic units) as the outcome variable was run to determine the relationship between age and occupational noise scores in the exposed group. The model showed that occupational noise exposure scores increased significantly as a function of age, *R* ^2^ = .326, *F*(1, 134) = 64.8, β = .052, *p* < .0001.

### Effects of Occupational Noise Exposure and Age on SPiN

#### Results of Group Comparisons

**Figure 2.**
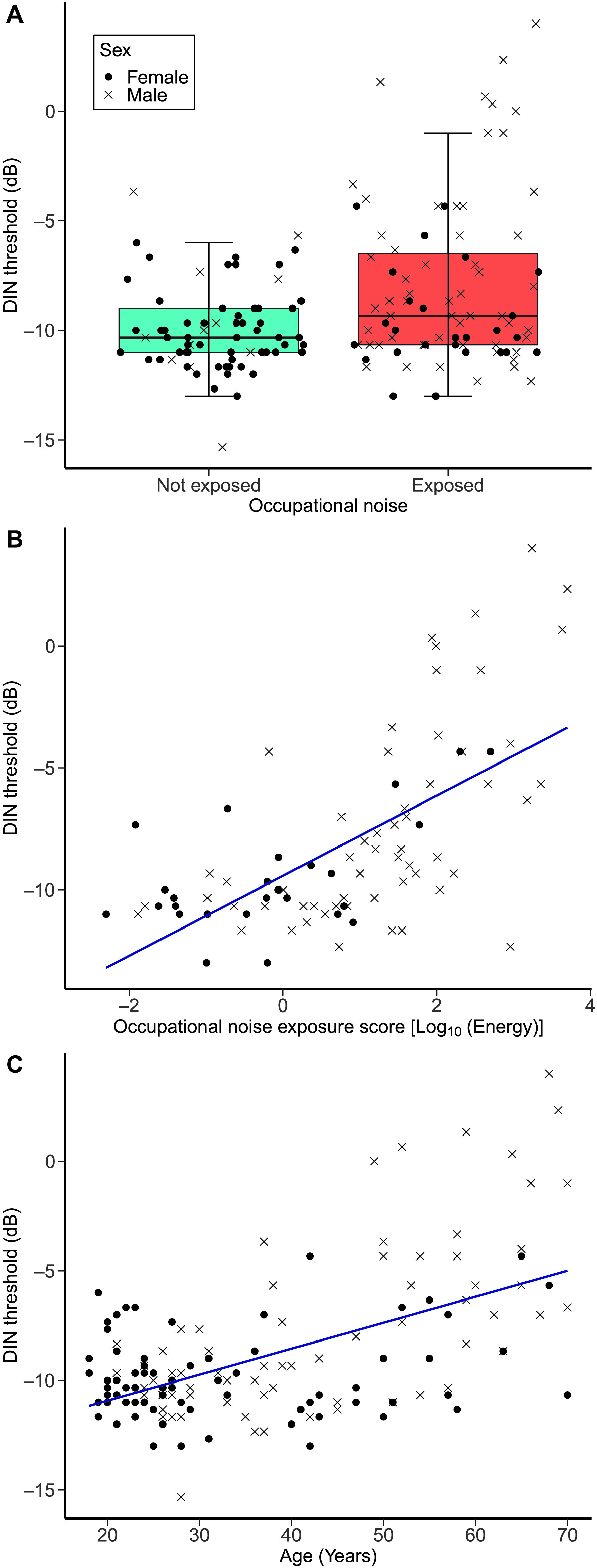
Digits-in-noise (DIN) thresholds. (A) The distribution of DIN thresholds as a function of the occupational noise exposure groups (not-exposed group, *n* = 69; exposed group, *n* = 83). (B) DIN thresholds as a function of occupational noise exposure scores in the exposed group. (C) DIN thresholds as a function of age across all study participants who completed the DIN task.

Figures 2A and 2C illustrate the distribution of DIN thresholds (given all participants who completed the DIN task; *n* = 152) across both occupational noise groups and as a function of participants' age, respectively. The first regression model (adjusted *R* ^2^ = .391), which considered all study participants who completed the DIN task, showed that the DIN thresholds of the exposed group (*n* = 83, *M* = −8.08 dB, *SD* = 3.77 dB, 95% CI [−8.90, −7.26]) were not significantly different from those of the not-exposed group (*n* = 69, *M* = −9.95 dB, *SD* = 1.95 dB, 95% CI [−10.41, −9.48]; *F*(1, 151) = 0.262, β = .04, *p* = .609), after controlling for the covariates. The same model showed that DIN thresholds significantly increased with increasing age, *F*(1, 151) = 33.15, β = .435, *p* < .0001, an effect that survived correction for multiple comparisons. Academic attainment was a significant predictor, in that higher academic attainment was associated with lower DIN thresholds, *F*(1, 151) = 17.8, β = −.305, *p* < .0001. The other covariates of recreational noise exposure, forward and backward digit span scores, and sex were not significant predictors.

#### Results for the Exposed Group

Figure 2B shows the DIN thresholds of the exposed group as a function of occupational noise exposure scores. The second regression model, which included participants of the exposed group only (adjusted *R* ^2^ = .475), showed that DIN thresholds increased as a function of higher occupational noise exposure, *F*(1, 82) = 7.84, β = .349, *p* = .007. Although pronounced, this effect did not survive Bonferroni–Holm correction for the 16 multiple comparisons used in the study. The same model showed that DIN thresholds significantly increased with increasing age, *F*(1, 82) = 13.62, β = .393, *p* < .0001, an effect that survived correction for multiple comparisons. The covariates of recreational noise exposure, forward and backward digit span scores, academic attainment, and sex were not significant predictors.

### Effects of Occupational Noise Exposure and Age on Self-Reported Hearing Ability

#### Results of Group Comparisons

**Figure 3.**
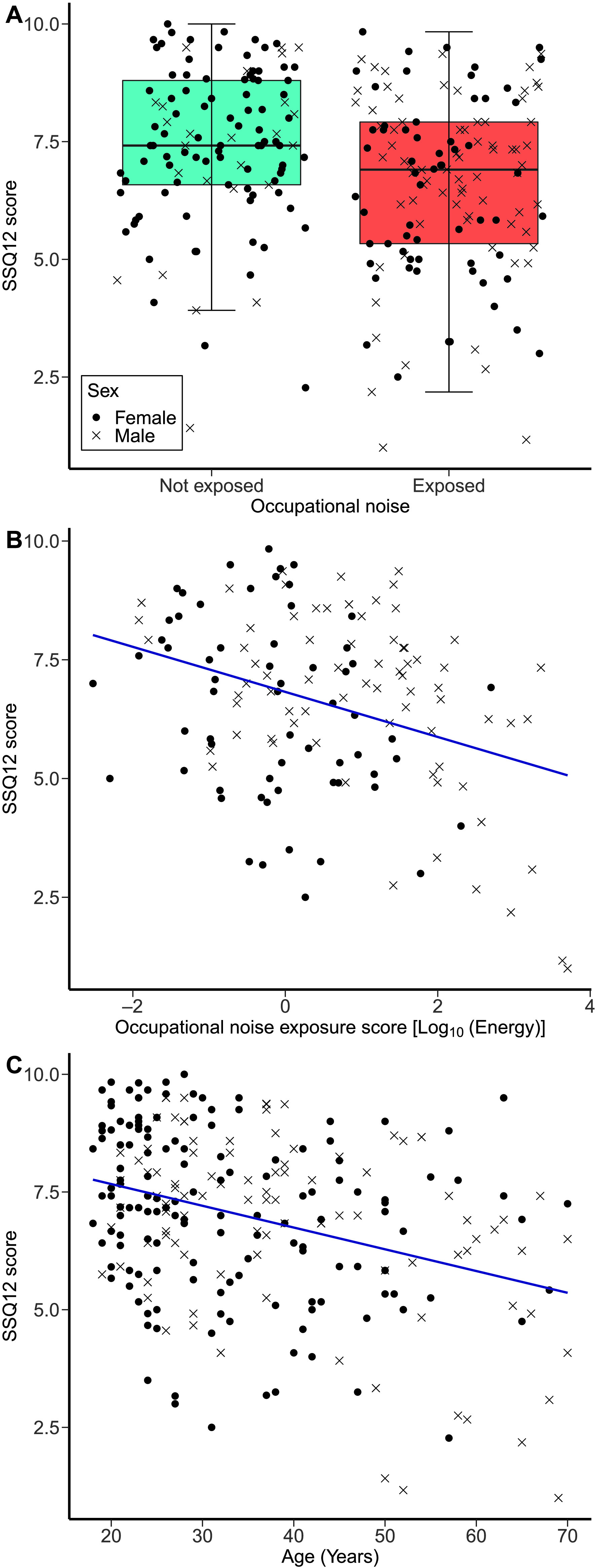
Speech, Spatial and Qualities of Hearing Scale (SSQ12) scores. (A) The distribution of SSQ12 scores as a function of the occupational noise exposure group (not-exposed group, *n* = 115; exposed group, *n* = 136). (B) SSQ12 scores as a function of occupational noise exposure scores in the exposed group. (C) SSQ12 scores as a function of age across all study participants.

Figures 3A and 3C show the distribution of SSQ12 scores (given all study participants) across both occupational noise groups and as a function of participants' age, respectively. The first linear regression model (adjusted *R* ^2^ = .136), which considered all study participants, showed that the SSQ12 scores of the exposed group (*n* = 136, *M* = 6.6, *SD* = 1.89, 95% CI [6.28, 6.92]) were lower than those of the not-exposed group (*n* = 115, *M* = 7.41, *SD* = 1.67, 95% CI [7.10, 7.72], *F*(1, 250) = 6.43, β = −.162, *p* = .012). However, this result did not survive correction for multiple comparisons. The same model showed that the SSQ12 scores significantly decreased with increasing age, *F*(1, 250) = 21.97, β = −.303, *p* < .0001, an effect that survived correction for multiple comparisons. The covariates of recreational noise exposure, forward and backward digit span scores, academic attainment, and sex were not significant predictors.

#### Results for the Exposed Group

Figure 3B shows the SSQ12 scores of the exposed group as a function of occupational noise exposure scores. The second linear regression model (adjusted *R* ^2^ = .176), which included participants of the exposed group only, showed that the SSQ12 scores decreased as occupational noise exposure increased, *F*(1, 135) = 5.78, β = −.279, *p* = .018. However, this result did not survive correction for multiple comparisons. The same model showed that the SSQ12 scores decreased with increasing age, *F*(1, 135) = 5.31, β = −.228, *p* = .023, an effect that did not survive correction for multiple comparisons. Sex was a significant predictor, in that being male was associated with worse SSQ12 scores, *F*(1, 135) = 7.78, β = −.249, *p* = .006. The other covariates of recreational noise exposure, forward and backward digit span scores, and academic attainment were not significant predictors.

### Effects of Occupational Noise Exposure and Age on Tinnitus

#### Results of Group Comparisons

**Figure 4.**
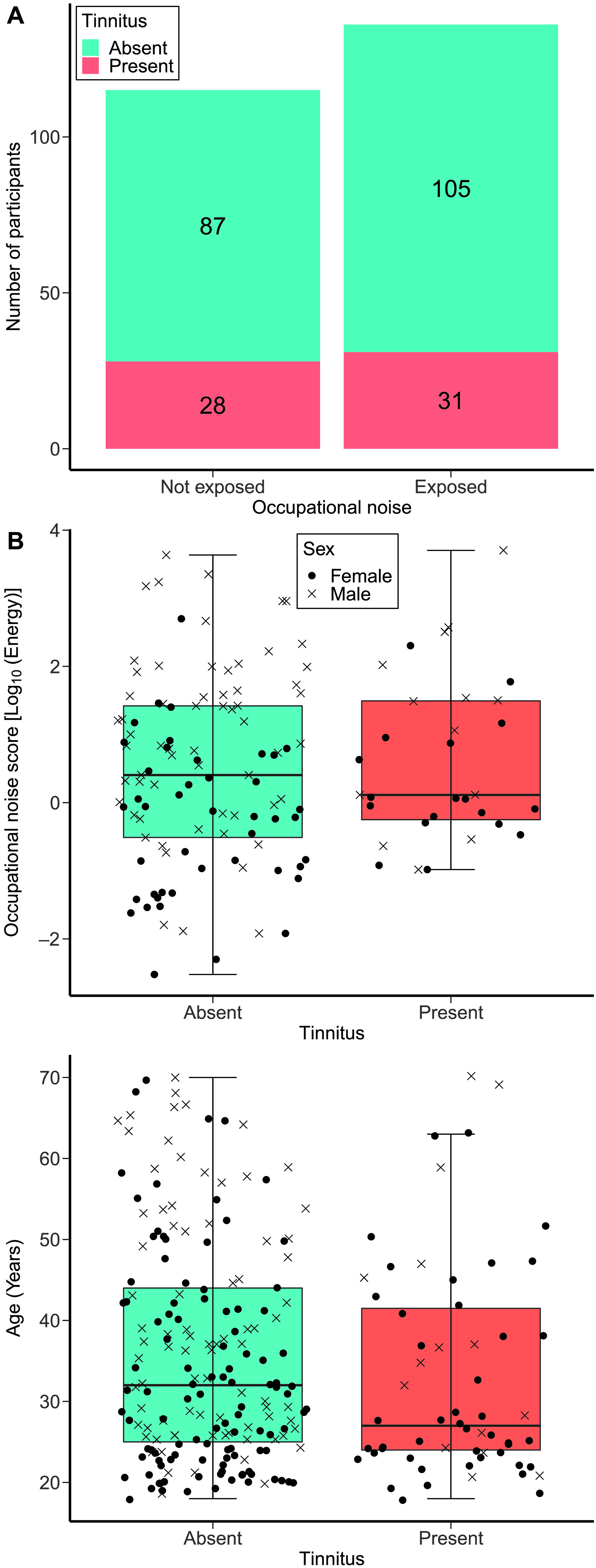
Tinnitus. (A) The number of participants with tinnitus as a function of the occupational noise group (not-exposed group, *n* = 115; exposed group, *n* = 136). (B) The distribution of occupational noise scores as a function of the presence of tinnitus (absent, *n* = 105; present, *n* = 31) in the exposed group. (C) The distribution of age as a function of the presence of tinnitus across all study participants.

Figure 4A illustrates the number of participants who reported tinnitus in both occupational noise groups, whereas Figure 4C shows the distribution of age as a function of the presence of tinnitus. In both figures, the outcomes across all study participants are shown. The first logistic regression model, which considered all study participants, showed that the proportion of participants with tinnitus was statistically similar across both occupational noise groups, *OR* = 0.82, 95% CI [0.43, 1.55], *p* = .534. Moreover, the same model showed that the proportion of participants with tinnitus did not vary significantly as a function of age, *OR* = 0.99, 95% CI [0.96, 1.01], *p* = .333. Sex was a significant predictor, in that being male was associated with a higher risk of tinnitus, *OR* = 0.439, 95% CI [0.217, 0.889], *p* = .022. The other covariates of recreational noise exposure, forward and backward digit span scores, and academic attainment were not significant predictors.

**Figure 5.**
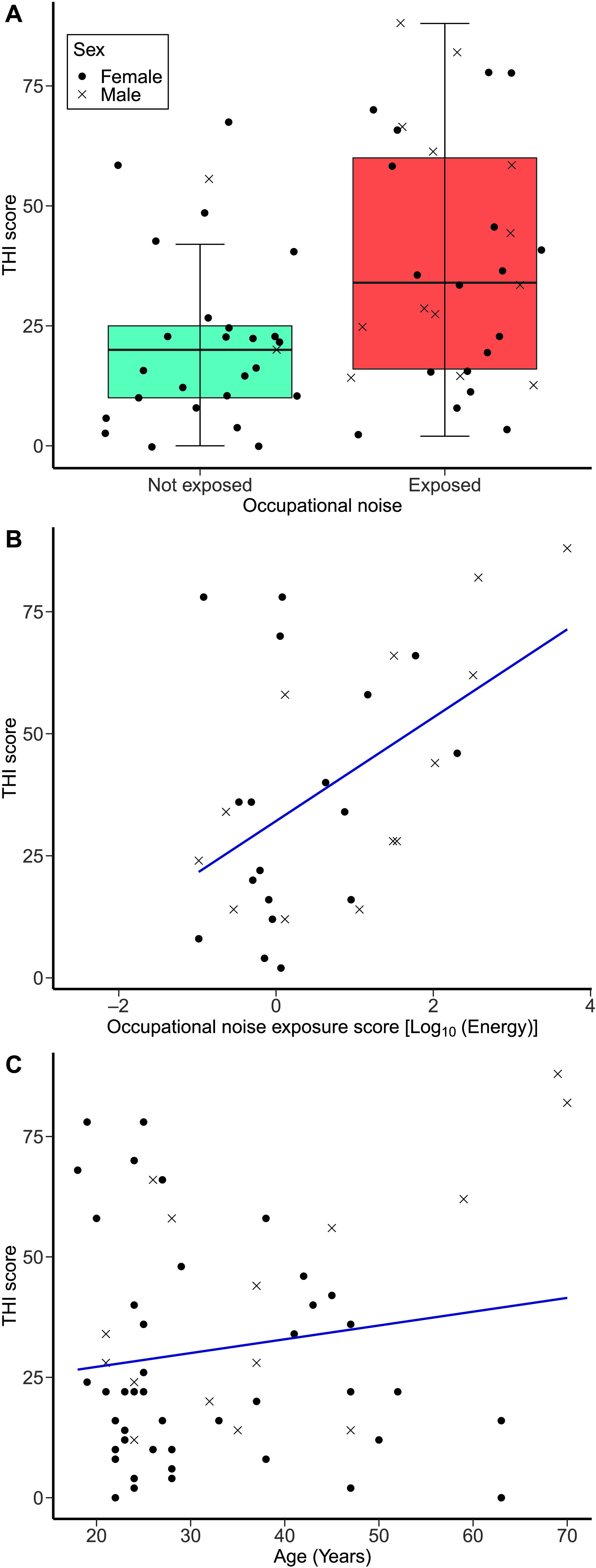
Tinnitus Handicap Inventory (THI) scores. (A) The distribution of THI scores as a function of the occupational noise group (not exposed group, *n* = 28; exposed group, *n* = 31). (B) THI scores as a function of occupational noise exposure scores in the exposed group. (C) THI scores as a function of age across all participants who completed the THI.

Figures 5A and 5C illustrate the distribution of THI scores (given all participants who completed the THI) across both occupational noise groups and as a function of age, respectively. The first exploratory regression model (adjusted *R* ^2^ = .083), *F*(1, 58) = 3.58, which considered all participants who completed the THI, showed that the THI scores of the exposed group (*n* = 31, *M* = 38.58, *SD* = 25.52, 95% CI [29.22, 47.94]) were statistically similar to those of the not-exposed group (*n* = 28, *M* = 22.22, *SD* = 18.40, 95% CI [14.94, 29.50]; *F*(1, 58) = 3.58, β = .254, *p* = .064). The same model showed that age did not predict THI scores, *F*(1, 58) = 1.258, β = .175, *p* = .267. The covariates of recreational noise exposure, forward and backward digit span scores, academic attainment, and sex were not significant predictors.

#### Results for the Exposed Group

Figure 4B shows the distribution of occupational noise exposure scores across participants with and without tinnitus in the exposed group. The second logistic regression model, which involved the participants of the exposed group only, showed that the proportion of participants with tinnitus increased with increasing occupational noise exposure, *OR* = 1.92, 95% CI [1.17, 3.14], *p* = .010. However, this result did not survive correction for multiple comparisons. The same model showed that age predicted a higher proportion of participants with tinnitus, *OR* = 0.95, 95% CI [0.915, 0.992], *p* = .018. This age effect did not survive correction for multiple comparisons. Sex was a significant predictor, in that being male predicted a higher risk of tinnitus, *OR* = 0.358, 95% CI [0.134, 0.956], *p* = .04. The covariates of recreational noise exposure, forward and backward digit span scores, and academic attainment were not significant predictors.

Figure 5B shows the THI scores of the exposed group as a function of occupational noise exposure. The second exploratory linear regression model (adjusted *R* ^2^ = .25) showed that THI scores increased as a function of occupational noise exposure, *F*(1, 31) = 6.14, β = .553, *p* = .021. The same model showed that age was not a significant predictor of THI scores, *F*(1, 31) = 0.073, β = .058, *p* = .789. The covariates of recreational noise exposure, forward and backward digit span scores, academic attainment, and sex were not significant predictors.

### Effects of Occupational Noise Exposure and Age on Hyperacusis

#### Results of Group Comparisons

**Figure 6.**
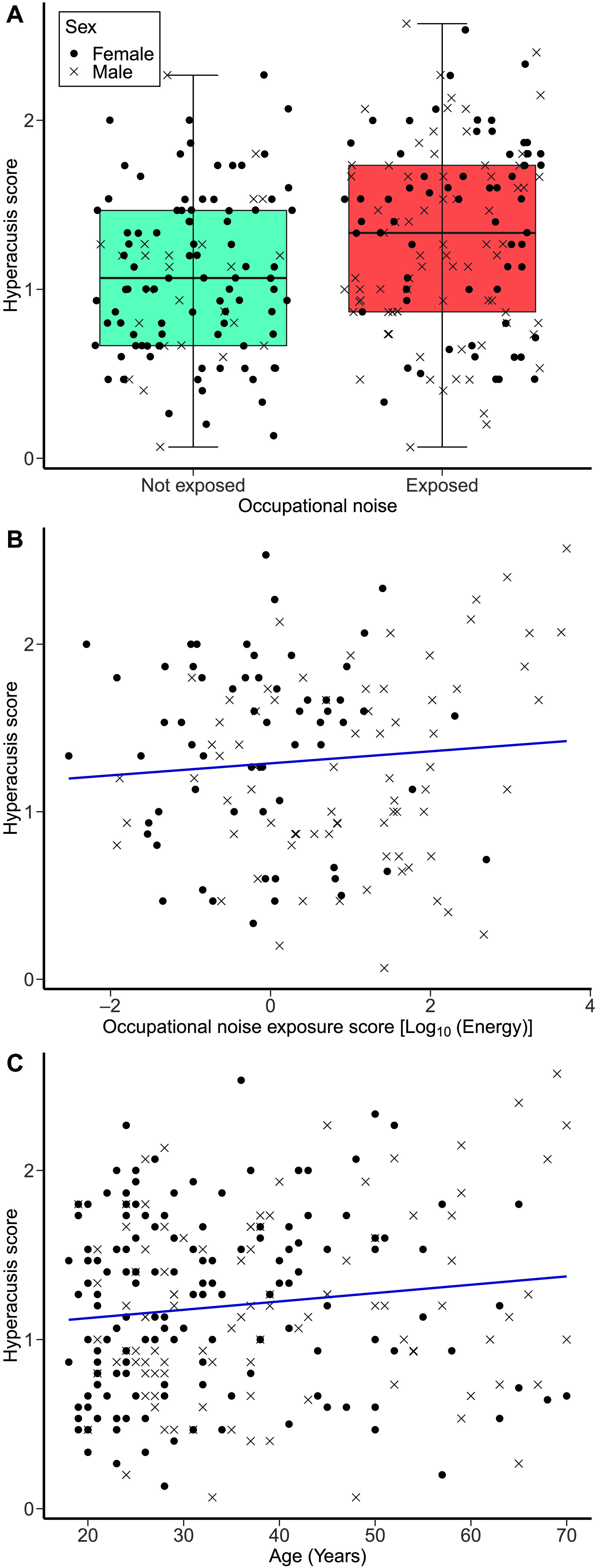
Hyperacusis scores. (A) The distribution of hyperacusis scores as a function of the occupational noise group (not-exposed group, *n* = 115; exposed group, *n* = 136). (B) Hyperacusis scores as a function of occupational noise exposure scores in the exposed group. (C) Hyperacusis scores as a function of age across all study participants.

Figures 6A and 6C show the distribution of hyperacusis scores (given all study participants) across both occupational noise groups and as a function of participants' age, respectively. The first regression model (adjusted *R* ^2^ = .053), which considered all study participants, showed that the hyperacusis scores of the exposed group (*n* = 136, *M* = 1.31, *SD* = 0.55, 95% CI [1.21, 1.40]) were significantly higher than those of the not-exposed group (*n* = 115, *M* = 1.08, *SD* = 0.47, 95% CI [0.99, 1.16]; *F*(1, 250) = 11.05, β = .223, *p* = .001). The effect survived correction for multiple comparisons. The same model showed that the hyperacusis scores did not vary significantly as a function of age, *F*(1, 250) = 1.90, β = .094, *p* = .169. The covariates of recreational noise exposure, forward and backward digit span scores, academic attainment, and sex were not significant predictors.

#### Results for the Exposed Group

Figure 6B shows the hyperacusis scores of the exposed group as a function of occupational noise exposure scores. The second regression model (adjusted *R* ^2^ = .027), which included participants of the exposed group only, showed that hyperacusis scores did not vary significantly as a function of occupational noise exposure (model adjusted *R* ^2^ = .027), *F*(1, 135) = 1.86, β = .172, *p* = .175. The same model showed that age did not predict worse hyperacusis scores, *F*(1, 135) = 0.137, β = .172, *p* = .712. Sex was a significant predictor, in that being male was associated with worse hyperacusis scores, *F*(1, 135) = 4.39, *p* = .038. The other covariates of recreational noise exposure, forward and backward digit span scores, and academic attainment were not significant predictors.

### Additional Exploratory Analyses

In the secondary analyses, occupational noise group (i.e., exposed and not exposed), age, and an interaction term (Occupational Noise Group × Age) were included as predictor variables in a model for each of the primary and secondary outcome variables. The covariates of sex, cognitive function (as reflected by the forward and backward digit span scores), academic attainment, and recreational noise exposure scores were included in all the models. Observed main effects were of (a) occupational noise group on DIN thresholds (model adjusted *R* ^2^ = .423), *F*(1, 151) = 13.16, β = −.45, *p* = .013; (b) academic attainment on DIN thresholds (model adjusted *R* ^2^ = .43), *F*(1, 151) = 7.36, β = −0.26, *p* < .0001; (c) age on SSQ12 scores (model adjusted *R* ^2^ = .13), *F*(1, 250) = 7.95, β = −.035, *p* = .005); and (d) sex on tinnitus presence, *OR* = 0.364, 95% CI [0.17, 0.78], *p* = .009. The interaction between occupational noise group and age was significant for DIN thresholds, *F*(1, 151) = 10.15, β = .659, *p* = .002, ɳ^2^_p_ = .066, such that the effect of noise exposure increased with increasing age. No other effects were significant.

In further exploratory analyses, the relations between the different continuous outcome variables were investigated to gain insights into potential correlations between them. Table 2 shows Spearman rho correlations between the different primary and secondary outcome measures with the number of participants (*n*) and the two-tailed significance level (*p* value) for each correlation comparison. For the correlation between tinnitus presence and the other outcome variable, the point biserial correlation coefficient is presented.

As shown in Table 3, the DIN speech recognition thresholds are significantly negatively correlated with the SSQ12 scores and positively correlated with the hyperacusis and THI scores. Moreover, the SSQ12 scores were found to be negatively correlated with tinnitus presence, hyperacusis, and THI scores. The tinnitus presence was significantly positively correlated with hyperacusis scores. Finally, the hyperacusis scores were significantly positively correlated with the THI scores.

**Table 3.** The Spearman rho and (for tinnitus presence) the point biserial correlation coefficients for the relationship between the different primary and secondary outcome measures.

Outcome measure	DIN (SRT)	SSQ12	Tinnitus presence	Hyperacusis	THI
DIN (SRT)	—	*r* = −.35**	*r* = −.07	*r* = .20*	*r* = .48**
		*p* < .0001	*p* = .368	*p* = .015	*p* = .003
		*n* = 152	*n* = 152	*n* = 152	*n* = 35
SSQ12	*r* = −.35**	—	*r* = −.19**	*r* = −.55**	*r* = −.41**
	*p* < .0001		*p* = .003	*p* < .0001	*p* = .001
	*n* = 151		*n* = 251	*n* = 251	*n* = 58
Tinnitus presence	*r* = −.07	*r* = −.19**	—	*r* = .217**	N/A
	*p* = .368	*p* = .003		*p* = .001	
	*n* = 152	*n* = 251		*n* = 251	
Hyperacusis	*r* = .20*	*r* = −.55**	*r* = .217**	—	*r* = .48**
	*p* = .015	*p* < .0001	*p* = .001		*p* < .0001
	*n* = 151	*n* = 251	*n* = 251		*n* = 58
THI	*r* = .48**	*r* = −.414**	N/A	*r* = .48**	—
	*p* = .003	*p* = .001		*p* < .0001	
	*n* = 35	*n* = 58		*n* = 58	

*Note.* The sample size (*n*) and significance level (*p* value) are presented for each comparison. DIN = digits-in-noise; SRT = speech recognition threshold; SSQ12 = Speech, Spatial and Qualities of Hearing Scale; THI = Tinnitus Handicap Inventory; N/A = not applicable.

* *p* < .05.

** *p* < .01 (uncorrected).

## Discussion

We hypothesized that occupational noise exposure and aging are associated with (a) poorer SPiN ability as reflected by higher DIN thresholds, (b) worse self-reported hearing ability as shown by lower SSQ12 scores, (c) higher prevalence of tinnitus as demonstrated by a higher proportion of participants reporting tinnitus, and (d) greater severity of hyperacusis as shown by higher hyperacusis scores and worse tinnitus handicap. Occupational noise exposure was associated with higher DIN thresholds, lower SSQ12 scores, greater hyperacusis scores, and a higher proportion of participants with tinnitus. However, except for hyperacusis severity, these effects did not survive strict (familywise error) correction for multiple comparisons. Increasing age was significantly associated with higher DIN thresholds and greater SSQ12 scores (after correction for multiple comparisons), but not with the presence of tinnitus, tinnitus handicap, or hyperacusis scores.

Our data showed a strong statistically significant correlation between occupational noise scores and age. This is in line with the outcome of Prendergast et al. (2019) who found that self-report lifetime noise exposure (expressed in logarithmic units) is significantly correlated with age (age range: 18–60 years; *r* = .50). In contrast, other studies that investigated the effects of self-report lifetime noise exposure and age failed to identify such a link (Carcagno & Plack, 2021; Shehabi, Prendergast, Guest, & Plack, 2022). A possible explanation for the discrepancy in findings may relate to limitations in noise exposure estimation tools used across the different studies, which could lack sensitivity to cultural, health, and lifestyle differences. The noise exposure questionnaire (based on the NESI) was translated from English into MSA but was not validated. Moreover, cumulative occupational noise exposure in Palestinian workers may increase as a function of age because workers are often present in noisy environments for many years over their life span with minimal hearing protection, as is the case in Palestine. Therefore, as these workers get older, their cumulative occupational noise levels increase accordingly. This pattern may not be seen when studying recreational noise exposure, as people may not necessarily be constantly exposed to such noises throughout their life span. Rather, an individual's recreational noise history may be dominated by exposures during their youth (e.g., bars, nightclubs, and earphones). Furthermore, noise exposures due to these factors may have been more common in the lifestyles of recent generations.

### SPiN

#### Effects of Occupational Noise Exposure on SPiN

SPiN ability as reflected by the DIN thresholds was similar across both occupational noise groups. In the current study, we hypothesized that occupational noise exposure may damage cochlear OHCs, IHCs, and synapses that connect IHCs with the auditory nerve. This is thought to decrease the audibility and intelligibility of speech signals at moderately loud suprathreshold levels and thus could result in poorer SPiN performance.

The lack of difference across both occupational noise groups with regard to DIN thresholds in the current study is consistent with the findings of other studies that investigated the effect of occupational noise on SPiN performance in audiometrically normal adults. For instance, Yeend et al. (2017) reported that audiometrically normal young and middle-aged adult musicians (who are typically exposed to high occupational noise throughout their career) performed similarly to nonmusicians on two different SPiN tasks: the Listening in Spatialized Noise-Sentences high-cue condition and the National Acoustics Laboratories Dynamic Conversations Test. The authors controlled for cognitive ability, EHF thresholds, and musical training. Similarly, Couth et al. (2020) showed that audiometrically normal musicians and nonmusicians, as well as participants deemed to have high noise and low noise exposures in both groups, had statistically similar CRM thresholds. Several other studies that examined SPiN performance as a function of lifetime noise exposure (i.e., including both occupational and recreational noise exposure) failed to show any compelling evidence for poorer SPiN performance secondary to increased lifetime noise exposure (Carcagno & Plack, 2021; Guest, Munro, et al., 2018; Prendergast, Millman, et al., 2017; Shehabi, Prendergast, Guest, & Plack, 2022; Valderrama et al., 2018).

Some explanations have been proposed for the lack of association between occupational/recreational noise exposure and SPiN performance. For instance, noise-induced CS with minimal OHC loss (i.e., no apparent audiometric threshold elevation) may result in a limited extent of low- and medium-SR ANF loss (Furman et al., 2013; Schmiedt et al., 1996). Thus, SPiN performance may be minimally affected in the absence of a significant OHC loss.

We also found that greater occupational noise exposure predicted higher (i.e., worse) DIN thresholds in the exposed group. However, this association did not survive correction for multiple comparisons. The worse SPiN performance, observed in the exposed group, as a function of higher exposure to occupational noise is possibly a consequence of undiagnosed NIHL. This is a very likely scenario, especially given the poor enforcement of hearing-related health and safety regulations in Palestine and the lack of awareness of the health risks associated with occupational noise hazards (International Labour Office, 2017, 2018; Schokry, 2015). Jaber et al. (2015) found that about 45% of male workers (*n* = 259) across 42 stone-saw workshops in the West Bank of Palestine were found to exhibit NIHL as measured by the standard pure-tone audiometry. The authors reported that the occupational noise levels in the stone-saw workshops ranged between 93 and 123 dB(A) L_eq_ for 8 hr per day (six working days a week; 48 working hours a week). This exceeds the safe limits of daily noise exposure of 88 dB(A) L_eq_ as proposed by Shaikh (1999) for occupational noise exposure for 8 hr a day for six working days a week. Hence, it is possible that the increased DIN thresholds in the current study may be correlated with elevated pure-tone audiometric thresholds at 2, 3, 4, and 6 kHz, as the data of Jansen et al. (2014) have shown. Thus, it is possible that several participants with occupational noise exposure in the current study may have had NIHL but were never formally diagnosed.

The worse DIN performance as a function of higher occupational noise exposure in the exposed group is consistent with the outcomes of some studies that investigated the SPiN ability of audiometrically normal workers of different professions. For instance, Kumar et al. (2012) reported that young and middle-aged train drivers with normal audiometric profiles exhibited poorer speech recognition scores (using custom sentences embedded in multitalker babble noise) compared to an age-matched control group. Vijayasarathy et al. (2021) reported similar outcomes in that a group of construction workers with normal hearing had significantly worse SPiN scores (using bisyllabic words embedded in speech-shaped background noise) relative to an age-matched control group with minimal noise exposure. Similarly, Hope et al. (2013) reported that audiometrically normal male Royal Air Force (RAF) pilots exhibited significantly worse SPiN thresholds (using the vowel–consonant–vowel test in International Collegium for Rehabilitative Audiology noise) compared to a control group of RAF administrators (with low exposure to occupational noise) with normal hearing.

Vijayasarathy et al. (2021) and Hope et al. (2013) employed relatively small sample sizes in their studies and that Kumar et al. (2012), Vijayasarathy et al., and Hope et al. did not correct the familywise error rate for multiple comparisons in their SPiN analyses. Thus, the significant SPiN outcomes reported in these studies may not survive correction for multiple comparisons.

We found a significant effect of increasing occupational noise exposure on DIN thresholds for the exposed group (the second regression model), but only a nonsignificant trend for the effect of some exposure versus no exposure (i.e., the first regression model). This difference may be explained by the nature of the relation between exposure and SPiN performance. Participants with low occupational noise exposure (say, ≤ 1.0 logarithmic units of occupational noise scores, forming a significant part of the exposed group) had generally similar performance compared to participants of the not-exposed group, as can be inferred from Figures 2A and 2B. In contrast, participants with high occupational noise exposure (i.e., > 2.0 logarithmic units of occupational noise scores) exhibited markedly higher DIN thresholds (see Figure 2B). It may be that a little occupational noise exposure has limited effects on SPiN, which deteriorates only after exposure that is more substantial. Thus, the second regression model may have had sufficient high-noise participants to show an occupational noise effect on DIN thresholds, whereas the first regression model lacked the necessary statistical power, due to reliance on an “exposed” group containing a relatively low proportion of the substantially exposed participants who drive the effect.

Consistent with this interpretation, it is worth highlighting that the current study involved many more participants with high noise exposure scores (> 2.0 logarithmic units of occupational noise) than previous studies that quantified SPiN ability and used the NESI to assess noise exposure (Couth et al., 2020; Guest, Munro, et al., 2018; Prendergast et al., 2019; Prendergast, Millman, et al., 2017; Shehabi, Prendergast, Guest, & Plack, 2022). These studies did not document any significant effects of noise exposure on SPiN ability. Thus, significantly worse DIN thresholds may become evident only after a certain level of cumulative lifetime noise exposure is reached. It is likely that, in the current study, participants with the highest occupational noise exposure exhibited undiagnosed peripheral auditory damage that manifested as markedly poorer DIN performance, whereas participants with little-to-moderate occupational noise exposure had much less noise-induced auditory damage. Thus, the effects of little-to-moderate occupational noise exposure on SPiN performance may not be clearly detectable by the DIN task used in the current study.

#### Effects of Age on SPiN

Higher DIN thresholds were significantly associated with older age in both regression models. These findings are in line with the outcomes of several lab-based studies that documented poorer SPiN thresholds as a function of older age in audiometrically normal or near-normal adults (Babkoff & Fostick, 2017; Carcagno & Plack, 2021; Füllgrabe et al., 2015; Johannesen et al., 2019; Patro et al., 2021; Prendergast et al., 2019). Recently, Shehabi, Prendergast, Guest, and Plack (2022) employed a similar online version of the DIN task to evaluate age-related differences in SPiN performance among British English adults with no past diagnosis of hearing impairment. The authors also found significantly higher DIN thresholds in the older group compared to the young group.

The increase in DIN thresholds with increasing age found in this study could be attributed to several age-related factors. First, age-related hearing threshold elevations, which were not measured, may result in worse SPiN thresholds (Hoben et al., 2017; Keithley, 2020; Wang et al., 2021; Yeend et al., 2019). Second, age-related CS and IHC-ANF loss, which have been confirmed to take place in otologically normal older adults (Viana et al., 2015; Wu et al., 2019, 2021), may cause poorer SPiN performance. Third, it is possible that age-related deficits in central auditory processing contributed to the observed age-related differences (Caspary et al., 2008; Ouda et al., 2015).

### Self-Reported Hearing Ability

#### Effects of Occupational Noise Exposure

Self-reported hearing ability, as expressed by the SSQ12 scores, was negatively associated with occupational noise exposure across both regression analyses. However, these effects did not survive correction for multiple comparisons. This trend of poorer self-reported hearing function among workers is similar to that reported by Kamerer et al. (2022), who found that greater history of impulsive noise exposure (e.g., explosion or firearm) significantly predicted lower SSQ12 scores in audiometrically normal adults (*n* = 111) aged 19–74 years. Similarly, Worede et al. (2022) who surveyed a group of metal and wood Ethiopian workers with exposure to unsafe levels of occupational noise found that about 20.7% of these workers believe they may have a hearing impairment. In line with these findings, John et al. (2018) showed that 41.5% of workers in gas-fired electric plants in Tanzania (*n* = 160) reported difficulties understanding conversations, whereas 53.8% of them mentioned that they may have a hearing loss.

Some studies failed to show an association between lifetime noise exposure and self-reported ability in adults with normal hearing. For instance, Yeend et al. (2017) found similar SSQ12 scores across two groups of audiometrically normal musicians and nonmusicians. Similarly, neither Carcagno and Plack (2021) nor Prendergast, Millman, et al. (2017) found a link between lifetime noise exposure and the SSQ12 and SSQ scores, respectively, among audiometrically normal/near-normal young and middle-aged adults. Recently, Shehabi, Prendergast, Guest, and Plack (2022), who employed a similar online approach, found that lifetime noise exposure did not predict SSQ12 scores in either age group (i.e., young vs. older adults). It is possible that the aforementioned studies failed to show a correlation between noise exposure and SSQ/SSQ12 scores because they involved audiometrically normal/near-normal adults. Thus, the SSQ/SSQ12 questionnaire may not be sensitive enough to detect the subtle differences (due to noise exposure) in hearing performance among individuals with normal hearing. In the current study, poorer self-reported hearing as a function of higher occupational noise exposure may be attributable in part to undiagnosed NIHL.

#### Effects of Age on SSQ12

Aging was associated with lower (i.e., worse) SSQ12 scores across both regression models. Only in the first model (which included all study participants) did the effect survive correction for multiple comparisons. Banh et al. (2012) reported that older adults with moderate sensorineural hearing loss exhibited significantly worse SSQ12 scores compared to younger adults with normal hearing. Moreover, older adults with normal hearing thresholds up to 4 kHz were found to have slightly (but insignificantly) higher SSQ scores compared to their younger counterparts, possibly due to age-related high-frequency sensorineural hearing loss (Banh et al., 2012). Therefore, the age-related decrease in SSQ scores observed in the current study could have been driven by the presence of older participants with undiagnosed age-related hearing impairments.

In contrast to our findings, other studies have observed no significant effect of aging in audiometrically normal/near-normal older adults on self-reported hearing ability using the SSQ and SSQ12 (Carcagno & Plack, 2021; Füllgrabe et al., 2015). Recently, Shehabi, Prendergast, Guest, and Plack (2022) found that young and older British adults without a past diagnosis of hearing impairment performed similarly on an online version of the SSQ12 questionnaire. The authors of the aforementioned studies suggested that the SSQ/SSQ12 might not be sensitive enough to establish the effect of aging on self-reported hearing function in audiometrically normal/near-normal adults. As discussed earlier, this is consistent with the possible presence in our sample of older adults with at least mild-to-moderate undiagnosed ARHL.

The low levels of awareness of age-related hearing impairment and the lack of appropriate audiology services in Palestine could be the main factors that explain why several Palestinian adults may reach older age with potentially undiagnosed and untreated age-related hearing difficulties. Recently, Harsha et al. (2019) showed that 21.1% of older Palestinians living in the West Bank and the Gaza Strip aged 60–69 years had some type of disability versus a rate of disability of 56.7% among those aged 80 years and above. These data, which were obtained from a nationally representative database, suggest a higher prevalence of disability among older adults compared to other developing nations (Harsha et al., 2019). Hearing impairment is likely one of these age-related disabilities that influence the quality of life of older adult Palestinians. According to the Palestinian Central Bureau of Statistics, the prevalence of adults who self-classify to have a significant hearing disability (defined as severe-to-profound hearing difficulty) across both the West Bank and the Gaza strip is 0.7% of the total population (Palestinian Central Bureau of Statistics, 2020). About 30% of these adults attribute their significant hearing disability to aging (Palestinian Central Bureau of Statistics, 2020). In 2018, the WHO estimated the global prevalence of DHI at 6.12%, whereas the prevalence of DHI in the Middle East and North Africa was noted to be 3.17% (WHO, 2018). The lower reported prevalence of DHI in Palestine and the Middle East compared to the global prevalence of DHI may be attributed to a large extent to social, cultural, and healthcare policy factors including the low of awareness of hearing impairment, as well as the lack of national policies that promote hearing health and the poor provision and access to ear and hearing services (WHO, 2018).

### Tinnitus

#### Effects of Occupational Noise Exposure on Tinnitus and Tinnitus Handicap

The occupational noise group (i.e., not exposed vs. exposed) did not predict the number of participants with tinnitus in the first logistic regression model that involved all study participants. In contrast, the number of participants with tinnitus increased with increasing occupational noise exposure for the exposed group. However, this effect does not survive correction for multiple comparisons.

Evidence from several studies suggests that unsafe occupational noise exposure is associated with a higher prevalence of tinnitus among workers of different ages and with normal and abnormal hearing levels (J. M. Bhatt et al., 2016; Couth et al., 2019; Dias et al., 2006; Fredriksson et al., 2015; Jafari et al., 2022; Masterson et al., 2016; Phoon et al., 1993; Ralli et al., 2017; Ringen et al., 2022). As discussed earlier, it is likely that some participants in the exposed group, especially those with the highest occupational noise exposure scores, had some degree of undiagnosed NIHL. NIHL, which typically manifests as elevated hearing thresholds secondary to OHC loss, is thought to be strongly associated with tinnitus (Boger et al., 2016; Dias et al., 2006; Kang et al., 2021; Mrena et al., 2007; Yankaskas, 2013).

As discussed earlier in relation to SPiN performance, it is possible that the first regression model (with all study participants) did not detect the hypothesized tinnitus effects due to its group-comparison design and the composition of its “exposed” group. Exposure to substantial occupational noise may be required before clear alterations in tinnitus prevalence are observed, and although the exposed group contained some such participants, participants with little-to-moderate occupational noise exposure dominated it.

The evidence on the relationship between occupational/recreational noise exposure and tinnitus in adults with normal hearing is mixed as some studies reported an association between them (Bramhall et al., 2018; Degeest et al., 2014; Guest, Munro, Prendergast, et al., 2017), whereas others did not (Rubak et al., 2008; Valderrama et al., 2018). Using a similar methodology to the current study, Shehabi, Prendergast, Guest, and Plack (2022) compared the proportion of participants with and without tinnitus across two groups of participants with no past diagnosis of hearing impairment (i.e., a young adult group and an older adult group) as a function of lifetime noise exposure (including both occupational and recreational noise exposure). The authors found that lifetime noise exposure was associated with a higher proportion of participants with tinnitus in the young, but not in the older, group.

At a physiologic level, there is some evidence to suggest that noise-induced CS, in the absence of OHC loss, may result in a higher compensatory gain in the central auditory system, which may account for a higher risk of tinnitus in noise-exposed humans (Bramhall et al., 2018; Hickox & Liberman, 2014; Schaette & McAlpine, 2011; Valderrama et al., 2018). However, some studies failed to document any links between noise-induced CS and the hypothesized increased compensatory central gain theory and subsequently a higher occurrence of tinnitus in audiometrically normal-hearing adults (Grose et al., 2017; Guest, Munro, & Plack, 2017; Guest, Munro, Prendergast, et al., 2017; Prendergast, Guest, et al., 2017).

In our exploratory analyses, higher THI scores (i.e., more severe tinnitus handicap) were associated with higher occupational noise exposure in the second regression model (involving participants of the exposed group only). No association between occupational noise exposure and THI was found in the first regression model that compared THI scores across both noise groups. The THI scores ranged between slight (raw score: 0–16) and catastrophic (raw score: 78–100) in those participants who completed the instrument. It is worth highlighting that, since the THI was completed by the subset of participants who reported tinnitus, statistical power was lower than for the other primary outcome measures.

The pattern of greater severity of tinnitus handicap as a function of higher occupational noise exposure scores as shown by the second regression model is consistent with the findings of a few studies such as those by I. S. Bhatt (2018), Tong and Yeung (2017), and Jafari et al. (2022). On the other hand, the lack of association between the occupational noise exposure group and THI scores (as shown by the first regression model) is in line with the findings of Shehabi, Prendergast, Guest, and Plack (2022) and House et al. (2018). These attempts to link the severity of tinnitus handicap to occupational/recreational noise exposure, including the current study, involved a wide variety of subjects with different hearing levels. Thus, undiagnosed NIHL caused by occupational noise may be a determinant of tinnitus severity.

#### Effects of Age on Tinnitus and Tinnitus Handicap

Older age did not predict the number of participants with tinnitus in the first regression model (involving participants from both noise groups). However, the prevalence of tinnitus increased with increasing age in the second regression model, which involved participants of the exposed group only. This effect did not survive correction for multiple comparisons.

A higher risk of tinnitus is thought to be strongly associated with older age (Ahmad & Seidman, 2004; McCormack et al., 2016). This is because aging is typically linked to a greater risk of neurological conditions, mental health disorders such as anxiety and depression, and ARHL, which can be influenced by health and lifestyle factors such as noise and ototoxic exposures, alcohol consumption, and smoking (Ahmad & Seidman, 2004; H. J. Kim et al., 2015; McCormack et al., 2016; Nondahl et al., 2010). This may explain the nonsignificant trend of higher prevalence of tinnitus as a function of older age that we found across participants of the exposed group.

We expected to see an age effect on the prevalence of tinnitus in the first regression model that included participants from both noise groups. However, since this model included participants without occupational noise exposure (alongside the exposed group) who did not work in physically demanding labor, then these participants are less likely to have been exposed to work-related hazards compared to the participants of the exposed group. Therefore, the effect of age on the presence of tinnitus, which may be primarily driven by age-related health and lifestyle factors as discussed earlier, may have not been detected by the first regression model. It is worth highlighting that factors related to the recruitment criteria such as the exclusion of participants with ototoxic exposure, neurologic symptoms, head/neck traumas, and any ear-related medical conditions might have decreased the chances of observing clear and significant age-related trends in both regression models.

Regarding tinnitus severity, age did not predict THI scores in either exploratory linear regression model. However, tinnitus severity, annoyance, and handicap are thought to increase as a function of age, due to a higher risk of age-related comorbidities such as neurological and psycho-emotional disorders, higher cumulative exposure to noise and ototoxic substances, and worse overall health (I. S. Bhatt, 2018; J. M. Bhatt et al., 2016; Hiller & Goebel, 2006). The findings of the current study are consistent with several other studies that failed to find an association between aging and worse THI scores (Pinto et al., 2010; Ralli et al., 2017; Shehabi, Prendergast, Guest, & Plack, 2022; Udupi et al., 2013). A possible reason for this null finding is the exclusion of participants with possible age-related factors that may worsen tinnitus handicap such as cognitive decline, neurological conditions, psycho-emotional disorders, intake of ototoxic medications, and ear pathology. Moreover, since a subset of participants completed the THI instrument (i.e., those who reported tinnitus only), the THI regression models may lack the statistical power to detect the hypothesized age effects, if any, on THI scores.

### Hyperacusis

#### Effects of Occupational Noise Exposure on Hyperacusis

Higher occupational noise exposure significantly predicted worse hyperacusis severity in the first linear regression model (which included participants from both noise groups). However, no association between occupational noise exposure and hyperacusis scores was found in the second linear regression model (involving the participants of the exposed group only). It is worth highlighting that our further exploratory analyses showed a significant positive correlation between the presence of tinnitus and the severity of hyperacusis, which is in line with several pieces of evidence on the link between tinnitus and hyperacusis (Andersson et al., 2002; Baguley et al., 2013; Henry et al., 2014).

Several studies have found a clear association between occupational/recreational noise exposure and hyperacusis in young adults with normal hearing (Camera et al., 2019; Couth et al., 2020; Fredriksson et al., 2022; Jafari et al., 2022; Pienkowski, 2021; Shehabi, Prendergast, Guest, & Plack, 2022). The findings of these studies are similar to those of the current study. Undiagnosed occupational NIHL may help explain the increased severity of hyperacusis as a function of occupational noise exposure (Auerbach et al., 2014; Knipper et al., 2013; Pienkowski et al., 2014). Our further exploratory analyses showed a significant correlation between DIN thresholds (which may be affected by OHC loss) and hyperacusis scores. Moreover, even if occupational noise exposure did not produce large threshold elevations in the current study, potential noise-induced CS might result in an increased central auditory compensatory gain, which may lead to hyperacusis alongside tinnitus (Hickox & Liberman, 2014; Schaette & McAlpine, 2011). However, some studies have failed to find evidence for the central compensatory gain mechanism in audiometrically normal adults in relation to the generation of hyperacusis (Couth et al., 2020; Möhrle et al., 2019). Further research is necessary to confirm the effect of noise exposure on hyperacusis in adults with normal hearing.

Although we expected to observe worse hyperacusis severity as a function of higher occupational noise exposure in the second linear regression model, it is possible that this (with participants of the exposed group) had lower statistical power compared to the first model to detect the hypothesized effect. Another potential explanation for the discrepancy across both models is that the risk of worse hyperacusis severity may be noticeably increased only after exposure to at least some occupational noise. Then, the hyperacusis severity may not increase any further as a function of greater occupational noise exposure.

#### Effects of Age on Hyperacusis

Higher risk and prevalence of hyperacusis are typically seen among older adults (Andersson et al., 2002; Paulin et al., 2016; Smit et al., 2021). This is because aging often results in pathologic changes in the central and peripheral auditory systems including OHC, IHC, and ANF loss (Ouda et al., 2015; Schuknecht & Gacek, 1993; Wu et al., 2019, 2021). Moreover, age-related psycho-emotional and neurological comorbidities, which are thought to be linked to hyperacusis (Baguley, 2003), are typically more prevalent at an older age (Andersson et al., 2002; Paulin et al., 2016; Smit et al., 2021; Tyler et al., 2014). The data of the current study, however, showed no significant association between age and hyperacusis severity. These findings are in line with a similar recent online study by Shehabi, Prendergast, Guest, and Plack (2022) that reported no association between age group (i.e., young vs. older) of participants with no past diagnosis of hearing/memory impairments and the severity of hyperacusis.

The effect of age on hyperacusis severity may primarily be determined by the presence and combination of accompanying medical comorbidities, lifestyle factors, socioeconomic status, and overall health rather than age itself. Therefore, the current study might have missed the hypothesized effect on hyperacusis, since the majority of the participants were in good general health and did not have past diagnoses of hearing, neurological, or cognitive impairments.

## Strengths, Limitations, and Directions for Future Research

The current study has several strengths in terms of the novelty of the design and the population studied. The current data, which were collected from Palestinian workers who are typically exposed to unsafe levels of occupational noise, provide insights into a demographic that has rarely been considered in auditory research. Given the difficulties that are associated with the recruitment of at-risk workers in developed countries due to laws and regulations on hearing protection, the current study provides some unique insights into the effects of occupational noise exposure on the auditory system.

The online nature of the current study allowed the researchers to reach out and recruit a wide and large demographic of Palestinian workers from different socioeconomic backgrounds. This was due to the convenience and ease of online participation. Thus, this online approach enabled the testing of a well-represented sample with considerable statistical power. In addition, the self-report and behavioral instruments were novel in that these were forward and backward translated and verified into Arabic (by a registered translator). Moreover, these instruments were delivered through a user-friendly online platform that enabled participants to take part at their convenience using their personal and smart devices.

We acknowledge several limitations in our approach. First, it was not possible to measure the participants' audiometric thresholds to verify their hearing status. This meant that some participants might have had undiagnosed noise-induced or age-related hearing impairments, which could potentially have influenced the self-reported and behavioral outcomes. Nonetheless, we attempted to rule out possible confounds such as ototoxic exposures, neurological and cognitive impairments, and diagnosed hearing loss by excluding prospective participants who reported them.

Second, the current study heavily relied on self-reported questionnaires to generate predictor and outcome variable data such as occupational noise exposure, subjective hearing ability, tinnitus presence and handicap, and hyperacusis severity. A major limitation in the self-reported questionnaires is that they primarily depend on participants' ability to answer questions accurately and recall/imagine specific situations. The self-report of some participants may not have been accurate, and hence, this may decrease the confidence in the data. In addition, some of the instruments translated into or developed in Arabic were not tested for their validity and reliability.

Third, although the Arabic DIN test is a novel attempt to assess the SPiN performance of Arabic-speaking Palestinian participants, there may be uncontrolled intersubject variability in the data due to differences in the quality and bandwidth of the sound produced by the different brands of headphones/earphones employed by our participants. We attempted to reduce this variability by low-pass filtering both the digits and the background noise at a knee point of 8 kHz. This may minimize the effect of high-frequency regions, which exhibit the greatest performance differences across different headphone and earphone types. Finally, some participants may have performed the DIN test in reverberant or noisy environments, which could add further intersubject variability. However, in an attempt to minimize the risk of this confound, we clearly instructed our participants to attempt the DIN test in the quietest possible place with minimal reverberations and distractions.

## Conclusions

The data of the current study, which were derived entirely through online instruments, suggest that occupational noise exposure and age may be associated with worse SPiN performance and poorer self-reported hearing ability. Occupational noise exposure, but not age, predicted a higher prevalence of tinnitus and greater tinnitus handicap as well as greater severity of hyperacusis. Although many of the outcomes seen did not survive the strict correction for multiple comparisons, the effects of (a) occupational noise exposure on hyperacusis severity and (b) age on SPiN performance and self-reported hearing ability did persist after correction. Although there was no way to confirm the extent to which our results were influenced by undiagnosed NIHL and ARHL, the effects of unsafe occupational noise exposure and age seem to clearly affect the hearing function of adults in Palestine. Further lab-based research is necessary to verify the findings of the current study and present further evidence on the impact of occupational noise on worker populations with potentially unsafe exposures during their lifespan. These research efforts may help in encouraging the local authorities to implement hearing-related health and safety regulations in developing countries such as Palestine.

## Author Contributions

All authors listed have made a substantial, direct, and intellectual contribution to the work and approved it for publication.

## Data Availability Statement

The datasets presented in this study can be found online on the Open Science Framework repository (https://osf.io/k2cws).

## Ethics Statement

This study was approved by the University of Manchester Research Ethics Committee before the beginning of the data collection. Upon participation, participants provided their written informed consent.

## Supplementary Material

10.1044/2022_JSLHR-22-00461SMS1 Supplemental Material S1 The findings of the original multiple regression models for all primary and secondary outcome measures treating both occupational noise exposure and age as continuous predictor variables.

10.1044/2022_JSLHR-22-00461SMS2 Supplemental Material S2 Clinical and Demographic Questionnaire (Arabic)

10.1044/2022_JSLHR-22-00461SMS3 Supplemental Material S3 Noise exposure questionnaire (Arabic)

10.1044/2022_JSLHR-22-00461SMS4 Supplemental Material S4 SSQ12 Questionnaire (Arabic)

10.1044/2022_JSLHR-22-00461SMS5 Supplemental Material S5 Tinnitus handicap inventory (Arabic)

10.1044/2022_JSLHR-22-00461SMS6 Supplemental Material S6 Khalfa hyperacusis questionnaire (Arabic)

10.1044/2022_JSLHR-22-00461SMS7 Supplemental Material S7 The distribution of occupational noise exposure scores across all study participants.
