# Supplementary material for: Noise Exposure in Palestinian Workers Without a Diagnosis of Hearing Impairment: Relations to Speech-Perception-in-Noise Difficulties, Tinnitus, and Hyperacusis
Source: J Speech Lang Hear Res. 2023 Feb 20;66(3):1085–109. doi: 10.1044/2022_JSLHR-22-00461 (PMC10205103; doi:10.1044/2022_JSLHR-22-00461)
Supplement: Supplemental Material S1 [file JSLHR-66-1085-s001.pdf]

Supplemental Material S1. The findings of the original multiple regression models for all primary and secondary outcome measures treating both occupational noise exposure and age as continuous predictor variables.

**Outcome variable per regression model**	**Predictor variables**	**Adjusted R^2**	***F*(*df*)**	***p*-value**	**Beta**	**95%CI of Beta**
**DIN thresholds**	Occupational noise exposure	.426	9.052 (1,151)	.003**	—	—
	Age	.426	22.805 (1,151)	< .0001***	—	—
**SSQ12 scores**	Occupational noise exposure	.149	6.915 (1, 250)	.009**	—	—
	Age	.149	15.135 (1, 250)	< .0001***	—	—
**Tinnitus presence**	Occupational noise exposure	—	—	.045*	1.30	1.006–1.679
	Age	—	—	.129	0.979	0.953–1.006
**Hyperacusis scores**	Occupational noise exposure	.147	11.152 (1, 250)	.001**	—	—
	Age	.147	0.784 (1, 250)	.377	—	—
**THI scores**	Occupational noise exposure	.150	6.066 (1, 58)	.018*	—	—
	Age	.150	0.885 (1, 58)	.352	—	—

*$p < .05$. **$p < .01$. ***$p < .001$
