# Supplementary material for: Noise Exposure in Palestinian Workers Without a Diagnosis of Hearing Impairment: Relations to Speech-Perception-in-Noise Difficulties, Tinnitus, and Hyperacusis
Source: J Speech Lang Hear Res. 2023 Feb 20;66(3):1085–109. doi: 10.1044/2022_JSLHR-22-00461 (PMC10205103; doi:10.1044/2022_JSLHR-22-00461)
Supplement: Supplemental Material S2 [file JSLHR-66-1085-s002.pdf]

Supplemental Material S2

Clinical and Demographic Questionnaire (Arabic)

أستمارة البيانات الديموغرافية

في هذا القسم، نرجو منكم التكرم بالإجابة على بعض الأسئلة المتعلقة بالمعلومات الشخصية والإجتماعية والأسئلة الأخرى المرتبطة بحاسة السمع وبالحالة الصحية بشكل عام.

1- يرجى تزويدنا بالبريدالإلكتروني الخاص بكم ______________ (قد نقوم بالتواصل معكم بخصوص التحويلة الطبية أو نتيجة السحب على الجائزة أو للمشاركة في أبحاث مستقبلية في حال موافقتكم على ذلك).

2- (أختياري): يرجى تزويدنا برقم هاتفكم: _____________

3- الجنس:

أ- ذكر

ب- أنثى

ج- غير ذلك (حدد: __________).

4- العمر (حسب عيد ميلادك الأخير): ______________ (مثلا: 55 سنة)

5- هل كنت في موقف عرضك لأصوات عالية خلال ال24 ساعة الماضية (أصوات عالية بمستوى الأصوات في الأعراس)؟

أ- لا

ب- نعم (في حال أخترت نعم، الرجاء الإجابة على السؤال أدناه)

1- الرجاء تحديد نوعية الأصوات العالية التي تعرضت لها خلال ال24 ساعة الماضية؟ ___________

2- الرجاء تحديد عدد الساعات والدقائق (تقريبا) التي تعرضت فيها لهذه الأصوات خلال ال24 ساعة الماضية__________ (مثلا: 3 ساعات و30 دقيقة)

6- هل تعتقد أن لديك مشكلة سمع حاليا؟

أ- لا

ب- نعم (في حال أخترت نعم، الرجاء الإجابة على السؤال أدناه)

1- في أي أذن تعتقد أن لديك مشكلة سمع؟

أ- اليمين

ب- اليسار

ج- الأذنين معا

د- غير متأكد

7- هل تعتقد أن مستوى السمع لديك يتغير من يوم لآخر؟

أ- لا

ب- نعم، ويصاحبه شعور بالدوار.

ت- نعم، ولا يصاحبه شعور بالدوار

8- في حال أجبت بنعم على السؤال السابق، يرجى تزويدنا بالتفاصيل حول التغير في مستوى السمع من يوم لآخر (أختياري)

____

9- هل تعتقد أنك تعاني من دوار (دوخة) بشكل مزمن حاليا؟

أ- لا

ب- نعم (الرجاء الإجابة على السؤال أدناه في حال أختيار نعم)

الرجاء تحديد في السطر الأول متى بدأت تشعر بالدوار (الدوخة) من خلال تحديد الشهر والسنة الذي بدأت فيها أعراض الدوخة. وعلى السطر التاني الرجاء ذكر إن كانت الدوار (الدوخة) مرتبط بوضع صحي معين.

____

____

10- هل تعتقد أنك تعاني من الطنين حاليا؟ (الطنين هو الشعور بصوت داخلي في الأذن كالرنين أو الضجيج ولكن بدون وجود مصدر خارجي حقيقي للصوت)

أ- نعم

ب- لا

11- هل يوجد لديك أي حالة طبية مرضية أخرى أو إحتياجات خاصة محددة؟

أ- لا

ب- نعم (الرجاء تحديد أي الحالات الطبية لديك):

____

____

____

12- هل أنت مدخن؟

أ- لا

ب- نادرا

ت- أقل من 10 سجارات في اليوم الواحد

ث- أكثر من 20 سيجارة في اليوم الواحد

13- في حال أنك مدخن، الرجاء تحديد عدد السنوات (تقريبا) التي كنت مدخنا فيها: ____________ (مثلا 8 سنوات).

14- هل يوجد لديك أحد الأقارب في العائلة لديهم مشاكل في السمع (باستثناء ضعف السمع الناتج عن التقدم بالسن).

أ- نعم (الرجاء تحديد صلة القرابة: ______________)

ب- لا

ت- لا أعرف

ث- أفضل عدم الإجابة على هذا السؤال.

15- هل أصابك إلتهاب مزمن في أذنك الوسطى مؤخرا؟

أ- لا

ب- نعم (وتم علاجها باستعمال المضادات الحيوية)

ت- نعم (وتم علاجها جراحيا عن طريق وضع أنابيب التهوية على الطبلة)

ث- نعم (تم الشفاء منها بصورة طبيعية بدون الحاجة لعلاج طبي)

ج- نعم (ما زلت أعاني من الإلتهاب حتى الآن).

16- في حال أجبت بنعم على السؤال السابق، الرجاء تحديد الأذن التي حدث فيها الإلتهاب المزمن؟

أ- الأذن اليمين

ب- الأذن اليسار

ت- الأذنتين معا

ث- لا ينطبق

17- هل خضعت لعملية جراحية في الأذن في السابق؟

أ- لا

ب- نعم (الأذن اليمين)

ت- نعم (الأذن اليسار)

ث- نعم (الأذنتين معا)

18- في حال أنك أجبت على السؤال السابق بنعم، الرجاء تحديد متى قمت باجراء العملية على السطر الأول أدناه، وسبب العملية على السطر الثاني:

____

____

____

19- هل قمت بتعاطي أي من الأدوية التالية في السابق لمدة زمنية طويلة نسبيا (أسابيع-لأشهر)؟ (يرجى أختيار كل ما ينطبق)

- ☐ الأدوية المضادة للإلتهابات (بما يشمل الأسبيرين، مسكنات الآلام العادية وخافض الحرارة).
- ☐ أدوية العلاج الكيميائي الخاصة بعلاج مرض السرطان
- ☐ مضادات الحساسية
- ☐ الأدوية المضادة لمرض الملاريا.
- ☐ الأدوية المضادة للإكتئاب
- ☐ الأدوية المضادة للتوتر والقلق
- ☐ المضادات الحيوية

☐ لا شيء مما ذكر أعلاه.

20- في حال أنك قمت بأخذ أي من الأدوية المذكورة أعلاه، يرجى تحديدلكل نوع دواء قمت باختياره وتحديد المدة الزمنية التقريبية لإستخدامه (بالسنوات والأشهر) وحجم الجرعة وموعد أخذها (مثلا: يوميا أو 3 مرات أسبوعيا الخ..)

____

____

____

____

____

21- هل تشرب الكحول؟

أ- لا

ب- نعم (مرة واحدة شهريا أو أقل).

ت- نعم (2-4 مرات شهريا)

ث- نعم (2-3 مرات أسبوعيا)

ج- نعم (أكثر من 4 مرات أسبوعيا).

22-هل تعتقد أن لديك مشاكل في الذاكرة أو الإدراك؟

أ- لا

ب- نعم (الرجاء توضيح طبيعة هذه المشاكل باختصار : ______________________)

23- هل قمت بالعمل في مكان فيه ضجيج عال سابقا؟

أ- لا

ب- نعم (يرجى التأكد من إجابة سؤال 25)

24- في حال أجبت بنعم على السؤال السابق، هل كنت تستخدم سدادات حماية الأذن بشكل منتظم في مكان العمل الذي فيه ضجيج مرتفع؟

أ- نعم

ب- لا

ت- لا ينطبق

25- في حال أجبت بنعم على سؤال 23، الرجاء تحديد طبيعة العمل الذي تعرضت فيه للضجيج المرتفع:

____

26-ما هي أعلى درجة تحصيل علمي لديك؟

أ- مدرسة أبتدائية

ب- مدرسة إعدادية

ت- شهادة الثانوية العامة

ث- تعليم صناعي / مهني

ج- درجة البكالوريوس الجامعية

ح- دراسات جامعية عليا (ماجستير و دكتوراة).
