# Supplementary material for: Noise Exposure in Palestinian Workers Without a Diagnosis of Hearing Impairment: Relations to Speech-Perception-in-Noise Difficulties, Tinnitus, and Hyperacusis
Source: J Speech Lang Hear Res. 2023 Feb 20;66(3):1085–109. doi: 10.1044/2022_JSLHR-22-00461 (PMC10205103; doi:10.1044/2022_JSLHR-22-00461)
Supplement: Supplemental Material S3 [file JSLHR-66-1085-s003.pdf]

Supplemental Material S3

Noise exposure questionnaire (Arabic)

أستمارة التعرض للضجيج

الهدف من هذه الأستمارة هو تحديد مستوى تعرضك للضجيج خلال فترة حياتك. سنطلب منك تحديد النشاطات التي تعرضت فيها للضجيج، ومن ثم تحديد المدة الزمنية لكل نشاط ومستوى الضجيج فيه.

هذه الإستمارة مقسمة إلى أربعة أقسام:
قسم أ: التعرض للضجيج المرتبط بنشاطات مرتبطة بالعمل أو التعليم
قسم ب: التعرض للضجيج المرتبط بالنشاطات الترفيهية
قسم ج: التعرض للأصوات الصادرة من سماعات الأذن
قسم د: التعرض للضجيج الصادر من الأسلحة النارية

الرجاء الإجابة على كل الأسئلة في كل الأقسام، وذلك سواء بأختيار الإجابة الملائمة لك أو بتعبئة الفراغ بالمعلومات المطلوبة.

قسم أ: التعرض للضجيج المرتبط بنشاطات مرتبطة بالعمل أو التعليم
في هذا القسم، سنطلب منك معلومات مرتبطة بنشاطات تعرضت فيها للضجيج خلال العمل أو التعليم.
نقصد بالنشاطات التي فيها "ضجيج" هي الحالات التي تشعر بأنه كان عليك رفع مستوى صوتك للحديث مع شخص لديه مستوى سمع طبيعي ولا يقوم بارتداء سدادات الأذن الواقية و يبعد عنك مسافة 4 أقدام (أي متر و20 سم تقريبا).

خلال حياتك، كم نشاط فيه ضجيج خلال العمل أو التعليم كنت متواجدا فيه؟

الآن، سنقوم بالأستفسار عن أول نشاط فيه ضجيج كنت فيه خلال العمل أو التعليم

ماذا كان ذلك النشاط؟ _______________

هل كان النشاط فيه ضجيج عال لدرجة أنك تحتاج لرفع صوتك للحديث مع شخص يبعد عنك مسافة 4 أقدام (أي متر و20 سم تقريبا)؟

أ- نعم
ب- لا

أخبرنا كم من الوقت تقريبا تعرضت لهذا الضجيج، من خلال الإجابة على الأسئلة التالية على أسطر منفصلة.

السطر الأول: كم سنة تعرضت لهذا الضجيج؟

السطر الثاني: كم أسبوع في السنة (بالمعدل) تعرضت لهذا الضجيج؟

السطر الثالث: كم يوم في الأسبوع (بالمعدل) تعرضت لهذا الضجيج؟

السطر الرابع: كم ساعة في اليوم (بالمعدل) تعرضت لهذا الضجيج؟

الآن، أخبرنا كم كان مستوى هذا الضجيج مرتفعا بالمعدل:

في وجود هذا الضجيج، كيف كان عليك التحدث مع شخص لديه مستوى سمع طبيعي ولا يرتدي سدادات حماية الأذن و يبعد عنك مسافة 4 أقدام (أي 1 متر و20 سم) حتى يستطيع أن يسمعك؟

أ- التحدث بمستوى صوت طبيعي من مسافة 4 أقدام
ب- رفع الصوت لنبرة متوسطة من مسافة 4 أقدام
ت- الحديث بنبرة مرتفعة من مسافة 4 أقدام
ث- الحديث بنبرة مرتفعة جدا من على مسافة 4 أقدام
ج- الصراخ من مسافة 4 أقدام
ح- الصراخ من مسافة قدمين (أي 60 سم تقريبا)
خ- الصراخ في أذن الشخص بشكل مباشر

هل قمت بارتداء سدادات الأذن الواقية من الضجيج خلال هذا النشاط؟

أ- نعم
ب- لا

كم نسبة الوقت الذي قمت فيه بارتداء سدادات الأذن الواقية من الضجيج خلال هذا النشاط.

0% الى 100%

أدناه أنواع مختلفة من سدادات الأذن الواقية من الضجيج مرقمة من 1 إلى 7

الرجاء أختيار رقم سدادات الأذن الذي قمت بارتدائه في هذا النشاط؟

قسم ب: في هذا القسم، سنقوم بسؤالك عن تعرضك للضجيج في نشاطات ترفيهية خلال فترة حياتك

نقصد بالنشاطات التي فيها "ضجيج" هي الحالات التي تشعر بأنه كان عليك رفع مستوى صوتك للحديث مع شخص لديه مستوى سمع طبيعي ولا يقوم بارتداء سدادات الأذن الواقية و يبعد عنك مسافة 4 أقدام (أي متر و20 سم تقريبا).

نقصد بالنشاطات الترفيهية تلك التي مررت بها باستثناء تلك التي حدثت في العمل أو التعليم

الرجاء أختيار جميع الخيارات التي تنطبق عليك وفيها نشاطات ترفيهية تعرضت فيها للضجيج:

1- العروض الموسيقية التي فيها موسيقى مرتفعة
2- المهرجانات الموسيقية (بما يشمل الأعراس والمنسابات العامة)
3- النوادي الليلية والبارات
4- عزف الآلات الموسيقية أو الغناء
5- الأدوات الكهربائية (بما يشمل أجهزة العناية بالحدائق المنزلية)
6- أصوات المحركات (مثلا الدراجات النارية، والدراجات الرياضية)
7- أصوات مرتفعة مرتبطة بالرياضة (مثلا المباريات)
8- السينما
9- غير ذلك
10- لا شيء مما ذكر

قسم ج: التعرض للأصوات الصادرة من سماعات الأذن

في هذا القسم، سنقوم بسؤالك عن أستخدامك لسماعات الأذن وتعرضك للأصوات منها.

هل تستخدم / استخدمت سماعات الأذن للأستماع للأصوات؟

أ- نعم
ب- لا

هل تقوم بتحديد مستوى الصوت عند الاستماع من خلال سماعات الأذن على مستوى مرتفع نسبيا (أي 70% أو أعلى من الحد الأقصى لمستوى الصوت)؟

أ- نعم
ب- لا

أخبرنا كم من الوقت تقريبا تعرضت لهذه الأصوات، من خلال الإجابة على الأسئلة التالية على أسطر منفصلة.

السطر الأول: كم سنة تعرضت لهذا الضجيج؟

السطر الثاني: كم أسبوع في السنة (بالمعدل) تعرضت لهذا الضجيج؟

السطر الثالث: كم يوم في الأسبوع (بالمعدل) تعرضت لهذا الضجيج؟

السطر الرابع: كم ساعة في اليوم (بالمعدل) تعرضت لهذا الضجيج؟
الرجاء أعطاء المعلومات عن الأستماع للأصوات من خلال سماعات الأذن بحيث كان مستوى الصوت مضبوطا على مستوى مرتفع (أي أعلى من 70% من الحد الأقصى للصوت) فقط. الرجاء تجاهل أي حالات أخرى قمت بالإستماع فيها لمستوى صوت أقل من ذلك.

الرجاء تحديد مستوى الصوت (بالمعدل) الذي قمت بالاستماع عليه بشكل عام من خلال سماعات الأذن من خلال تحديد نسبة الصوت كنسبة مئوية من الحد الأقصى للصوت

70% إلى 100%

قسم د: التعرض للضجيج الصادر من الأسلحة النارية

في هذا القسم، سنقوم بسؤالك حول تعرضك للضجيج الصادر من الأسلحة النارية (الطلقات).

نحن بحاجة لمعلومات حول التعرض لأصوات الأسلحة النارية بدون أرتدائك لسدادات الأذن الواقية من الضجيج.

هل تعرضت في السابق لضجيج صادر من أسلحة نارية (مثلا بندقية، مسدس) بدون أن تكون مرتديا لسدادات الأذن الواقية من الضجيج؟

أ- نعم
ب- لا

ما هو نوع السلاح الناري الذي تعرضت لصوته؟

أ- رشاش مضاد للطائرات
ب- بندقية من عيار منخفض (0.17 أو 0.22)
ت- أي نوع آخر من البندقيات
ث- أي نوع آخر من الأسلحة النارية (مسدس، رشاش)

بالنسبة للضجيج الصادر من البندقية ذات العيار المنخفض، كم عدد الجولات التي تعرضت فيها لهذا النوع من الضجيج بدون استخدام سدادات الأذن الواقية؟ ____________________

بالنسبة للضجيج الصادر من البندقية ذات العيار المرتفع، كم عدد الجولات التي تعرضت فيها لهذا النوع من الضجيج بدون استخدام سدادات الأذن الواقية؟ __________________

بالنسبة للضجيج الصادر من الأسلحة النارية الأخرى (مثل المسدسات، الرشاشات)، كم عدد الجولات الي تعرضت فيها لهذا النوع من الضجيج بدون أستخدام سدادات الأذن الواقية؟ ____________________
