# Supplementary material for: Noise Exposure in Palestinian Workers Without a Diagnosis of Hearing Impairment: Relations to Speech-Perception-in-Noise Difficulties, Tinnitus, and Hyperacusis
Source: J Speech Lang Hear Res. 2023 Feb 20;66(3):1085–109. doi: 10.1044/2022_JSLHR-22-00461 (PMC10205103; doi:10.1044/2022_JSLHR-22-00461)
Supplement: Supplemental Material S4 [file JSLHR-66-1085-s004.pdf]

Supplemental Material S4

SSQ12 Questionnaire (Arabic)

أستمارة فهم الكلام و تمييز الأصوات وجودتها في المحيط

في هذه الإستمارة، تطرح الأسئلة التالية إستفسارات عن جوانب متعلقة بقدراتك السمعية وتجاربك في الاستماع للكلام في وضعيات مختلفة.

يرجى أختيار أجابة واحدة لكل سؤال من خيارات المتوفرة تحت السؤال. تتراوح الإجابات بشكل رقمي على الخيارات المتاحة (أرقام تتراوح بين 0 و 10) يعني إختيارك للرقم 10 أنك تستطيع أداء المهمة الموصوفة أو الشعور بها في السؤال بشكل ممتاز، بينما أخيار 0 يشير إلى أنك غير قادر على أداء المهمة أو الشعور بها. في حال كان أحد الأسئلة لا ينطبق يرجى أختيار (لا ينطبق).

1- أنت تقوم بالحديث مع شخص آخر بينما هناك تلفاز يعمل نفس الغرفة. هل تستطيع متابعة الحديث مع هذا الشخص وفهم ما يقول بدون الحاجة إلى خفض صوت التلفاز ؟

لا ينطبق 0 (لا، ابدا) ____________________ 10 (نعم، بشكل ممتاز)

2- تستمع لأحدهم وهو يتحدث إليك بينما هناك أخبار على التلفاز وأنت تحاول سماع وفهم الأخبار على التلفاز في نفس الوقت. هل تستطيع فهم حديث الشخص الذي يتحدث إليك وفهم كلام المذيع في التلفاز في نفس الوقت؟

لا ينطبق 0 (لا، ابدا) ____________________ 10 (نعم، أستطيع بشكل ممتاز)

3- أنت تقوم بإجراء حديث مع شخص واحد في غرفة بينما هناك أشخاص آخرون يقومون بالحديث فيما بينهم في نفس الغرفة. هل تستطيع متابعة الحديث مع هذا الشخص الذي يتحدث إليك وفهم كلامه؟

لا ينطبق 0 (لا، ابدا)____________________ 10 (نعم، أستطيع بشكل ممتاز)

4- أنت ضمن مجموعة مكونة من 5 أشخاص في مطعم مكتظ بالزبائن. أنت تستطيع أن ترى جميع أفراد المجموعة الذين معك. هل تستطيع متابعة الحديث ضمن سياق المجموعة وفهم المحادثة التي تجري بينكم؟

لا ينطبق 0(لا، ابدا)____________________ 10(نعم، أستطيع بشكل ممتاز)

5- أنت ضمن مجموعة من الأشخاص وهناك محادثة تجري بينكم بحيث أن الحديث ينتقل من شخص لآخر في هذه المحادثة. هل تستطيع متابعة الحديث وفهم الكلام بسهولة بدون أن يفوتك بداية الكلام عندما يقوم شخص ببدء الكلام في المجموعة؟

لا ينطبق 0(لا، ابدا)____________________ 10(نعم، أستطيع بشكل ممتاز)

6- أنت خارج المنزل وهناك كلب يقوم بالنباح بصوت عال. هل تستطيع تحديد موقع الكلب فورا، بدون محاولة النظر حولك لرؤية مكان الكلب؟

لا ينطبق 0(لا، ابدا)____________________ 10(نعم، أستطيع بشكل ممتاز)

7- هل تستطيع تحديد كم يبعد عنك باص أو شاحنة ما فقط من الصوت الصادر من المركبة وبدون الحاجة للنظر لتحديد الموقع؟

لا ينطبق 0(لا، ابدا) ____________________ 10(نعم، أستطيع بشكل ممتاز)

8- هل تستطيع تحديد إذا كان هناك باص أو شاحنة تقوم بالإقتراب أو الإبتعاد منك فقط من الصوت الصادر من المركبة دون الحاجة للنظر لتحديد موقعها؟

لا ينطبق 0(لا، ابدا)____________________ 10(نعم، أسطيع بشكل ممتاز)

9- عندما تقوم بالإستماع إلى أكثر من صوت في الوقت نفسه، هل تشعر بأن الأصوات تصبح متداخلة مع بعضها البعض؟

لا ينطبق 0(نعم، الأصوات متداخلة)______________ 10(لا، الأصوات غير متداخلة)

10-عندما تقوم بالإستماع إلى الموسيقى، هل تستطيع تمييز أصوات الآلات الموسيقية المختلفة الموجودة في الموسيقى؟

لا ينطبق 0(لا، ابدا) ____________________ 10 (نعم، أستطيع بشكل ممتاز)

11-هل تشعر بأن الأصوات اليومية التي تستطيع سماعها بسهولة تبدو واضحة لك (أي أنها غير مشوشة)؟

لا ينطبق 0(لا، غير واضحة ابدا) ______________ 10(نعم، واضحة تماما)

12-هل تشعر أن عليك التركيز بشدة عندما تقوم بالإستماع لشخص أو شيء ما؟

لا ينطبق 0(نعم، أنا بحاجة للتركيز بشدة) ____________ 10(لا، لا حاجة للتركيز)
