# Supplementary material for: Noise Exposure in Palestinian Workers Without a Diagnosis of Hearing Impairment: Relations to Speech-Perception-in-Noise Difficulties, Tinnitus, and Hyperacusis
Source: J Speech Lang Hear Res. 2023 Feb 20;66(3):1085–109. doi: 10.1044/2022_JSLHR-22-00461 (PMC10205103; doi:10.1044/2022_JSLHR-22-00461)
Supplement: Supplemental Material S5 [file JSLHR-66-1085-s005.pdf]

Supplemental material, Shehabi et al., "Noise Exposure in Palestinian Workers Without a Diagnosis of Hearing Impairment: Relations to Speech-Perception-in-Noise Difficulties, Tinnitus, and Hyperacusis," *JSLHR*, https://doi.org/10.1044/2022_JSLHR-22-00461

Supplemental Material S5

Tinnitus handicap inventory (Arabic)

ان الهدف من هذا الاستبيان هو تحديد المشاكل التي يتسبب بها طنين الاذن لديك. اختر "نعم" او "احيانا" او "كلا" لكل سؤال في المكان المناسب. نرجو منك ألا تتخطى اي سؤال. Xعبر وضع علامة

**1**. هل يصعب عليك التركيز بسبب الطنين؟	☐ نعم (4)	☐ أحيانا (2)	☐ كلا (0)
**2**. هل يؤثر مستوى الطنين على سمعك للناس؟	☐ نعم (4)	☐ أحيانا (2)	☐ كلا (0)
**3**. هل تشعر بالغضب بسبب الطنين؟	☐ نعم (4)	☐ أحيانا (2)	☐ كلا (0)
**4**. هل تشعر بالارباك بسبب الطنين؟	☐ نعم (4)	☐ أحيانا (2)	☐ كلا (0)
**5**. هل تشعر باليأس بسبب الطنين؟	☐ نعم (4)	☐ أحيانا (2)	☐ كلا (0)
**6**. هل تشكو كثيرا للناس حواليك من الطنين؟	☐ نعم (4)	☐ أحيانا (2)	☐ كلا (0)
**7**. هل تعاني من صعوبة بالنوم ليلاً" بسبب الطنين؟	☐ نعم (4)	☐ أحيانا (2)	☐ كلا (0)
**8**. هل تشعر انه ليس بإمكانك الهروب من الطنين؟	☐ نعم (4)	☐ أحيانا (2)	☐ كلا (0)
**9**. هل يحدّ الطنين من قدرتك على الأستمتاع بالانشطة الأجتماعيّة مثل الخروج لتناول العشاء أو الذهاب ألى السينما؟	☐ نعم (4)	☐ أحيانا (2)	☐ كلا (0)
**10**. هل تشعر بالاحباط نتيجة الطنين؟	☐ نعم (4)	☐ أحيانا (2)	☐ كلا (0)
**11**. هل تشعر بأنّ لديك مرض خطير بسبب الطنين؟	☐ نعم (4)	☐ أحيانا (2)	☐ كلا (0)
**12**. هل يصعب عليك الاستمتاع بالحياة نتيجة الطنين؟	☐ نعم (4)	☐ أحيانا (2)	☐ كلا (0)
**13**. هل يتداخل الطنين مع عملك او مسؤولياتك المنزلية؟	☐ نعم (4)	☐ أحيانا (2)	☐ كلا (0)
**14**. هل تشعر غالبا انك سريع الانفعال بسبب الطنين؟	☐ نعم (4)	☐ أحيانا (2)	☐ كلا (0)
**15**. هل تصعب عليك القراءة بسبب الطنين؟	☐ نعم (4)	☐ أحيانا (2)	☐ كلا (0)
**16**. هل يجعلك الطنين مستاءً ؟	☐ نعم (4)	☐ أحيانا (2)	☐ كلا (0)
**17**. هل يسبّب الطنين توتّر في علاقاتك مع بقيّة أفراد عائلتك و أصدقائك؟	☐ نعم (4)	☐ أحيانا (2)	☐ كلا (0)
**18**. هل تجد انه من الصعب عليك تركيز انتباهك على امور اخرى بعيدا عن الطنين؟	☐ نعم (4)	☐ أحيانا (2)	☐ كلا (0)
19. هل تشعر انه ليس لديك اي سيطرة على الطنين ؟	☐ نعم (4)	☐ أحيانا (2)	☐ كلا (0)
**20**. هل تشعر غالبا" بالتعب بسبب الطنين؟	☐ نعم (4)	☐ أحيانا (2)	☐ كلا (0)
**21**. هل تشعر بالكآبة بسبب الطنين؟	☐ نعم (4)	☐ أحيانا (2)	☐ كلا (0)
**22**. هل يدفعك الطنين الى القلق؟	☐ نعم (4)	☐ أحيانا (2)	☐ كلا (0)
**23**. هل تشعر بأنّك لم تعد قادر على التعايش مع الطنين؟	☐ نعم (4)	☐ أحيانا (2)	☐ كلا (0)
**24**. هل يسوء الطنين تحت الضغط؟	☐ نعم (4)	☐ أحيانا (2)	☐ كلا (0)
**25**. هل يشعرك الطنين بعدم الأمان؟	☐ نعم (4)	☐ أحيانا (2)	☐ كلا (0)
