# Supplementary material for: Noise Exposure in Palestinian Workers Without a Diagnosis of Hearing Impairment: Relations to Speech-Perception-in-Noise Difficulties, Tinnitus, and Hyperacusis
Source: J Speech Lang Hear Res. 2023 Feb 20;66(3):1085–109. doi: 10.1044/2022_JSLHR-22-00461 (PMC10205103; doi:10.1044/2022_JSLHR-22-00461)
Supplement: Supplemental Material S6 [file JSLHR-66-1085-s006.pdf]

Supplemental Material S6

Khalfa hyperacusis questionnaire (Arabic)

إستجواب خلفا الخاص بمرض فرط الحساسية للصوت

1. هل أنت تتعرض أو تعرضت للضوضاء ؟

2. هل تتأقلم مع الضوضاء بدرجة أقل من السنوات القليلة الماضية ؟

3. هل كان لديك يوما مشاكل فى السمع ؟ إن وجد فمن أى نوع ؟

3	2	1	0	
نعم كثير جدا	نعم كثير نوعا ما	نعم قليل	لا	
				1.هل أنت دائما تستخدم واقيات الأذن لتقليل الإحساس بالضوضاء ؟ "لا نأخذ فى الاعتبار إستخدام واقيات السمع فى حالات الضوضاء العاليه ".
				2.هل تجد صعوبه فى تجاهل الأصوات حولك فى مواقف الحياة العاديه ؟
				3.هل تجد صعوبه فى القراءه فى الضوضاء ؟
				4.هل تجد صعوبه فى التركيز فى الضوضاء ؟
				5.هل تجد صعوبه فى الاستماع للمحادثات فى الضوضاء ؟
				6.هل هناك شخص أنت تعرفه أخبرك أنك تتأقلم مع الضوضاء او أصوات معينه بطريقه سيئه ؟
				7.هل أنت حساس أو تتضايق من ضوضاء الشارع ؟
				8.هل تجد الضوضاء تضايقك فى بعض المواقف الاجتماعيه ؟
				9.هل إذا اقترح شخص ما فعل شئ مثلا الذهاب للسينما تبادر بالتفكير سريعا فى الضوضاء التى سوف تواجهها ؟
				10.هل ترفض دعوه أو ترفض الخروج بسبب الضوضاء التى سوف تواجهها ؟
				11.هل الضوضاء أو أصوات معينه تضايقك في الأماكن الساكنة أكثر من الأماكن التى بها ضوضاء بسيطه ؟
				12.هل الضغط العصبى والتعب يقللان من قدرتك على التركيز فى الضوضاء ؟
				13.هل تقل قدرتك على التركيز فى الضوضاء فى آخر اليوم ؟
				14.هل الضوضاء أو أصوات معينه تسبب لك ضغط نفسى أو تضايقك ؟
